# Genetic Susceptibility to Causal Relationship Between Iron Metabolism Disorder Involving Immunocytes and Risk of Pneumonia and Sepsis

**DOI:** 10.1002/fsn3.70422

**Published:** 2025-06-10

**Authors:** Zhenchao Wu, Taikang Yao, Zhongyu Han, Zilu Wang, Beibei Liu, Ming Lu, Jiajia Zheng, Ning Shen

**Affiliations:** ^1^ Department of Pulmonary and Critical Care Medicine Peking University Third Hospital Beijing People's Republic of China; ^2^ Department of Laboratory Medicine Peking University Third Hospital Beijing People's Republic of China; ^3^ Center for Infectious Diseases Peking University Third Hospital Beijing People's Republic of China

**Keywords:** immune cell, iron metabolism, Mendelian randomization analysis, pneumonia, sepsis

## Abstract

Recent studies showed ferritin increase and hemoglobin decrease to COVID‐19 severity and sepsis mortality. However, the potential relationship between iron metabolism disorders and susceptibility to pneumonia remains unclear. This study explores the association between iron metabolism disorder and susceptibility to bacterial pneumonia, viral pneumonia, and sepsis. GWAS data from the FinnGen and UK biobank is used for a two‐sample Mendelian randomization (MR) analysis, followed by MR meta‐analysis. Low serum iron levels were negatively associated with the risk of bacterial pneumonia (OR, 0.85; *p* = 0.04), influenza pneumonia (OR, 0.86; *p* = 0.03), and sepsis (OR, 0.81; *p* = 0.0004). Increased transferrin saturation and decreased total iron‐binding capacity were linked to higher risks of bacterial pneumonia and sepsis. Elevated liver iron content correlated positively with susceptibility to bacterial pneumonia (OR, 1.11; *p* = 0.007), influenza pneumonia (OR, 1.08; *p* = 0.03), and sepsis (OR, 1.13; *p* = 0.0007), but negatively with pneumococcal pneumonia (OR, 37.62; *p* = 0.0013). Neutrophils mediated the impact of serum iron and transferrin saturation on susceptibility to bacterial pneumonia and sepsis. This MR study confirms that disruptions in iron metabolism, including low serum iron levels and elevated liver iron content, increase susceptibility to bacterial and viral pneumonia as well as sepsis by affecting neutrophil function and cytokine levels. The findings emphasize the need for monitoring iron metabolism indicators in high‐risk populations and provide valuable insights for further mechanistic research and clinical intervention.

## Graphical abstract

This Mendelian randomization study demonstrates a U‐shaped relationship between iron homeostasis and infection risk, revealing that both iron deficiency and overload significantly increase susceptibility to bacterial pneumonia, viral pneumonia, and sepsis. Disruptions in systemic iron metabolism (e.g., low serum iron, elevated transferrin saturation) and intracellular iron accumulation impair neutrophil function and dysregulate cytokine responses, driving heightened infection severity. The findings emphasize the clinical need to monitor iron status in high‐risk populations and highlight iron homeostasis as a potential target for therapeutic intervention.

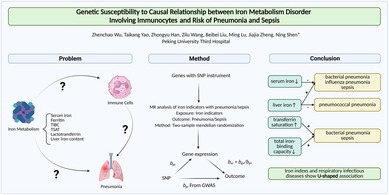

## 1 Introduction

The 2019 Global Burden of Disease Study data reveals that acute lower respiratory infections (LRIs), including pneumonia, affected 489 million people worldwide (GBD 2019 Diseases and Injuries Collaborators 2020) and has ranked the fourth leading cause of death (GBD 2017 Causes of Death Collaborators 2018). Pneumonia caused by bacteria or viruses accounts for the top two causes of LRI‐related deaths (Torres et al. 2021). According to the latest epidemiological surveys, *Streptococcus pneumoniae* and *influenza viruses* are the most common pathogens in community‐acquired pneumonia (CAP), holding their primary position in CAP whether before or after the COVID‐19 pandemic (Aliberti et al. 2021; Jain et al. 2015). Annually, about 1.19 million people die from pneumococcal pneumonia (GBD 2019 Diseases and Injuries Collaborators 2020), and 290,000 to 650,000 people die from influenza (Iuliano et al. 2018). Identifying early risk factors and predictive biomarkers for high‐risk populations on CAP remains an urgent issue. Once severe bacterial or viral pneumonia occurs, a large number of pathogens accompanied by toxins and inflammatory factors are released into the bloodstream, leading to sepsis (Cecconi et al. 2018; Gotts and Matthay 2016). Sepsis, a life‐threatening organ dysfunction caused by a dysregulated host response to infection, presents high disability and mortality rates, along with severe socioeconomic and public health burdens (Liu et al. 2014). Some studies showed that elevated ferritin, severe hemoglobin decline, malnutrition, and metabolic disorders are independent risk factors for acute infection patients to develop sepsis (Martin et al. 2006; Tolsma et al. 2014; Brandtner et al. 2020), suggesting that iron metabolism may play a crucial role.

In recent years, the critical role of iron in various life activities has garnered increasing attention. Besides being related to cardiovascular disease (Sawicki et al. 2023), chronic kidney disease (Allison 2023), and malignant tumors (Pasricha et al. 2021), it is also related to respiratory and infectious diseases (Tacke et al. 2016). The surge in research related to the COVID‐19 pandemic has revealed a significant association between ferritin levels and the severity of COVID‐19 prognosis (Mahroum et al. 2022; Chaubey et al. 2023). In our recent review (Wu et al. 2022), we systematically discussed: (1) Iron as a vital resource contested by humans and bacteria during infections, explaining the host–pathogen competition for iron, including the nutritional immunity mechanism (a defense mechanism that reduces serum iron levels to prevent pathogen proliferation and survival in order to prevent infection). (2) Common clinical indicators of iron metabolism, such as serum iron, ferritin, transferrin saturation (TSAT), and total iron‐binding capacity (TIBC), are considered potentially related to infectious anemia. (3) Host iron metabolism‐related regulatory factors, like LCN2 and hepcidin secreted by immune cells, which play significant roles in anti‐infection processes (Wu et al. 2022).

However, the role of iron metabolism disorders for acute pulmonary infections, apart from COVID‐19, caused by common pathogens remains unclear. Therefore, we aim to explore the role of iron metabolism levels in the susceptible risk, early identification, and severity assessment of pneumonia and sepsis, using Mendelian randomization (MR) to minimize confounding factors (Yao et al. 2023), and reveal the potential clinical associations between iron metabolism‐based nutritional immunity mechanisms and the development and progression of lung infections and sepsis.

Some parts of contents in this study had been presented at 2024 European Respiratory Society (ERS) Congress by oral presentation (No. OA5459).

## 2 Methods

### 2.1 Two‐Sample MR Study Design

We employed a bidirectional two‐sample MR design: a genetic instrumental variable analysis based on summary‐level data, with single nucleotide polymorphisms (SNPs) serving as instruments for risk factors. In one‐sample MR, weak instrument bias tends to align with observational associations due to shared chance correlations within the same dataset, while in two‐sample MR using independent samples, this bias is reduced and shifted toward the null because chance variations across datasets remain uncorrelated (Jiang et al. 2023). The two‐sample approach also mitigates the “winner's curse” bias that artificially underestimates causal effects in one‐sample analyses (Taylor et al. 2014). Additionally, two‐sample MR leveraging GWAS summary statistics improves statistical power, especially for studies with binary outcomes, by efficiently combining large‐scale genetic data (Burgess et al. 2015).

**FIGURE 1:**
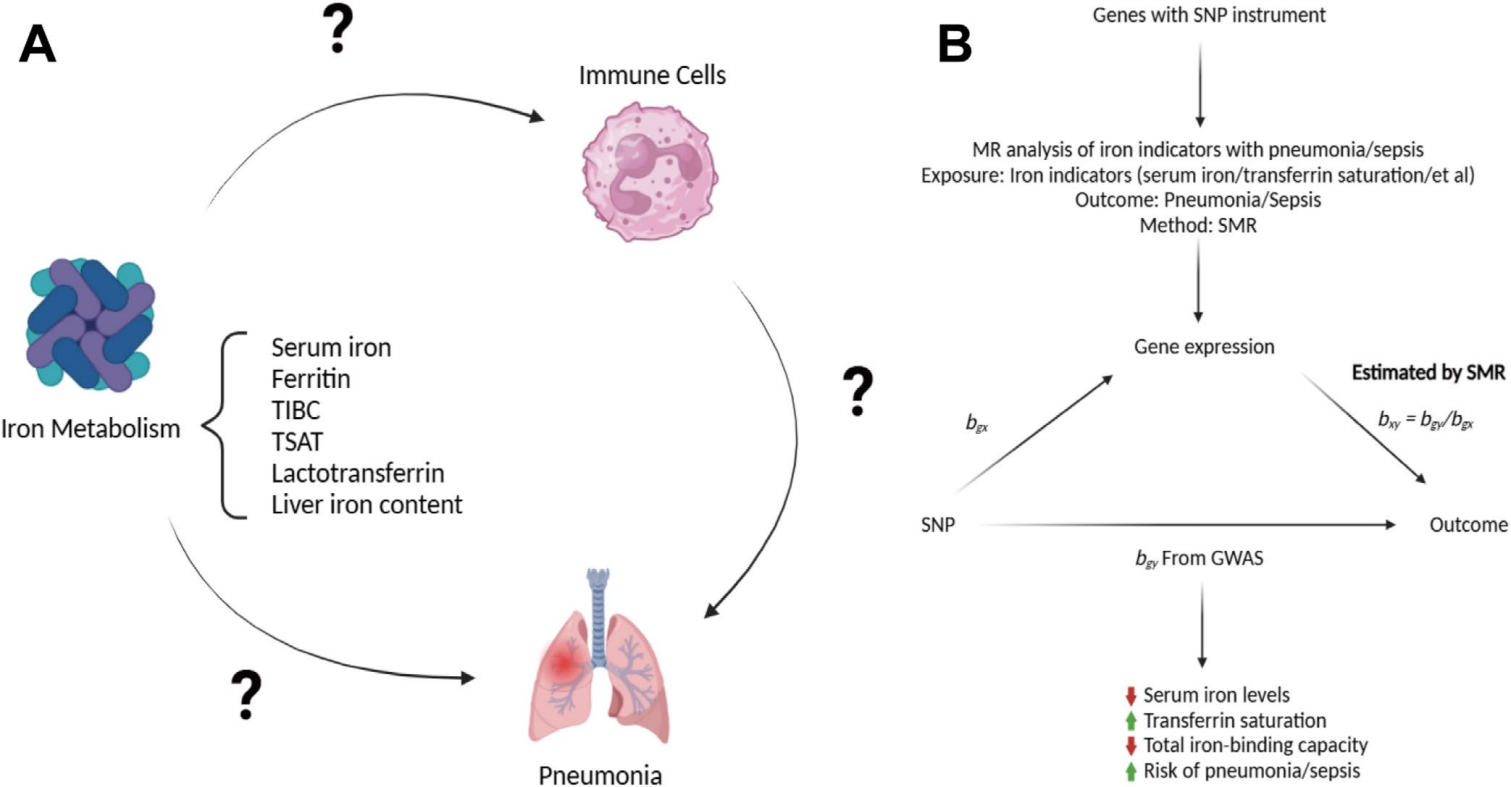
Summary of Mendelian randomization study design. GWAS, genome‐wide association studies; SMR, summary data‐based Mendelian randomization; SNP, single nucleotide polymorphism; TIBC, total iron‐binding capacity; TSAT, transferrin saturation.

Initially, we conducted a bidirectional two‐sample MR analysis to establish the causal relationships between iron metabolism levels and the susceptibility to bacterial pneumonia (*n* = 23,770), streptococcal pneumonia (*n* = 648), viral pneumonia (*n* = 1463), influenza (*n* = 4262), influenza pneumonia (*n* = 29,924), sepsis (*n* = 21,797), and high risk of 28‐day sepsis mortality (*n* = 2243). The causal relationships were quantified using inverse variance weighted (IVW), weighted median, and MR Egger methods, followed by meta‐analysis (Figure 1). Finally, we applied two‐step MR analysis to evaluate whether immune cells and cytokines act as causal mediators in the pathway between iron metabolism disorders and infection risk. All data used were publicly available and derived from the European population. We adhered to the STROBE‐MR reporting guidelines (Skrivankova et al. 2021).

### 2.2 Genetic Instruments Selection

The MR study comprises three components: the outcome (infectious diseases), the exposure hypothesized to causally relate to the outcome (iron status), and the genetic instruments for the exposure. On one hand, using SNPs as genetic instruments for the exposure, which are randomly allocated among individuals, allows MR analysis to serve as an alternative to randomized controlled trials (RCTs). On the other hand, our MR analysis is only valid if the following three assumptions are satisfied: (1) robust associations exist between the SNPs and the iron metabolism markers; (2) the SNPs are not associated with confounders of the relationship between the iron metabolism markers and infection; (3) the SNPs influence infection only through their effects on the iron metabolism markers (serum iron, ferritin, TIBC, TSAT, lactotransferrin, liver iron content), implying the absence of pleiotropy (Emdin et al. 2017). It should be particularly noted that these key iron metabolism biomarkers were selected as exposure factors following an extensive literature review, yet each exhibits inherent strengths and weaknesses in clinical practice. A detailed interpretation and critical reflection of integrating considerations are provided in Supporting Information.

We selected SNPs associated with levels of serum iron, ferritin, TSAT, and other iron metabolism markers from previous GWAS studies to serve as genetic proxies for exposure. The instrument‐outcome associations were estimated using the effect sizes of these same SNPs from GWAS conducted on pneumonia and sepsis datasets obtained from the FinnGen biobank. SNPs related to iron metabolism levels were derived from a meta‐analysis of three GWAS conducted in Iceland, the UK, and Denmark, involving levels of serum iron (*n* = 163,511), ferritin (*n* = 246,139), total TIBC (*n* = 135,430), and TSAT (*n* = 131,471) (Bell et al. 2021). Additionally, SNPs associated with serum lactoferrin levels were derived from 3301 individuals of European ancestry (Sun et al. 2018), SNPs associated with liver iron content from 32,858 individuals of European ancestry (Liu et al. 2021), SNPs associated with ferritin from 3301 individuals of European ancestry (Sun et al. 2018) SNPs associated with LCN2 from 3301 individuals of European ancestry (Sun et al. 2018), and SNPs associated with neutrophils and monocytes from a meta‐analysis of three European cohorts (Astle et al. 2016; Keller et al. 2014; Lin et al. 2017).

The levels (high, normal, or low) associated with each biomarker were determined based on the effects of their corresponding SNPs. For each biomarker, we identified multiple genome‐wide significant SNPs through MR analysis. The direction of effect for these SNPs was interpreted using their beta coefficients: SNPs with positive beta values (*β* > 0) were considered to indicate higher‐than‐normal status, while those with negative beta values (*β* < 0) indicated predisposition to lower‐than‐normal status. The overall biomarker classification was derived from the weighted sum of individual SNP effects.

The *F*‐statistic is a measure of instrument strength and is related to the proportion of phenotypic variance explained by genetic variation (*R* ^2^), sample size (*N*), and the number of instruments (*k*). The calculation formula is $F=R^{2}\left(N-k-1\right)/k\left(1-R^{2}\right)$ (Burgess and Thompson 2011). The $R_{i}^{2}$ for instrument *i* is calculated using the approximation $R_{i}^{2}=2\times {\mathrm{EAF}}_{i}\times \left(1-{\mathrm{EAF}}_{i}\right)\times \beta_{i}^{2}$, where EAF_*i*_ is the effect allele frequency, and $\beta_{i}$ is the estimated genetic effect on exposure (Meddens et al. 2021). Only genetic variations with an *F* statistic ≥ 10 are used, indicating a relatively low risk of instrument bias in the MR analysis (Palmer et al. 2012).

Genetic variants with *p* < 5 × 10^−6^ and linkage disequilibrium (LD) *r* ^2^ < 0.001 were selected as instrumental variables. Using PLINK clumping with a window size of < 10,000 kb, the identified SNPs were clumped for independence using European genomic data to obtain an independent set of SNPs (Davies et al. 2018). Genetic variants with *p* < 5 × 10^−6^ and linkage disequilibrium (LD) *r* ^2^ < 0.001 were selected as instrumental variables. This selection ensures that causal estimates are not biased by genetic variants that are strongly associated with other potential confounding factors, thereby revealing causal effects in the MR analysis. The tool selection process is illustrated in Figure 1.

### 2.3 Acquisition of GWAS Outcome

To obtain GWAS summary data sets related to lung infection and sepsis, we utilized the latest MRC Integrative Epidemiology Unit (IEU) GWAS database, which contains up to 50,037 GWAS summary data sets (v7.5.26, as of January 10, 2024). Specifically, we used data from FinnGen and the UK Biobank. Table S1 summarizes the demographic characteristics of the GWAS summary data sets used in the MR analysis.

### 2.4 Statistical Analysis

In the MR analysis, we performed a meta‐analysis of SNP‐specific Wald estimates using IVW with multiplicative random effects as needed. IVW regression was applied to test the potential causal relationship between iron metabolism levels and the risk of lung infection or sepsis, using SNPs associated with iron metabolism levels as instrumental variables. The final MR estimates of the relationship between iron metabolism levels and the risk of pneumonia or sepsis were based on SNP‐specific Wald estimates, which involve dividing the genetic association with lung infection by the genetic association with the genetic proxy for changes in iron metabolism levels (Palmer et al. 2011).

Since the IVW estimates may be biased if pleiotropic instrumental variables are introduced (Burgess et al. 2013), we conducted a series of sensitivity analyses to address pleiotropy in causal estimates. In the fixed‐effects variance‐weighted analysis, Cochran's *Q* statistic was calculated to quantify the heterogeneity produced by different genetic variants, with *p* ≤ 0.05 indicating the presence of pleiotropy (Bowden et al. 2017; Giri et al. 2020). If potential pleiotropy was detected, random‐effects IVW MR analysis was employed. Additionally, we used MR–Egger regression based on the intercept term to assess the potential presence of horizontal pleiotropy, with *p* < 0.05 considered as evidence of directional pleiotropy bias (Bowden et al. 2015). In the presence of horizontal pleiotropy, the slope coefficient from MR–Egger regression provides a consistent estimate of the causal effect.

We also conducted sensitivity analyses using the weighted‐median (Bowden et al. 2016) and weighted‐mode (Hartwig et al. 2017) methods under different assumptions. Furthermore, MR‐PRESSO was used to evaluate pleiotropy, identifying outliers with potential horizontal pleiotropy among multiple genetic variants (Verbanck et al. 2018). A leave‐one‐out analysis was performed to determine whether the results were biased or driven by a single SNP, thus assessing the impact of individual variants on the observed association.

All statistical analyses were conducted using R software (version 4.3.2) and the R package “TwoSampleMR” (version 0.4.26) (Hemani et al. 2018). A two‐sided *p*‐value < 0.05 was considered statistically significant.

### 2.5 Mediation Analysis

For significant MR associations, a two‐step MR analysis was applied to assess mediation effects (Figure S1). In the first step, genetic instruments for iron metabolism levels were used to estimate the causal effect of exposure on potential mediators. In the second step, genetic instruments for identified mediators were used to assess their causal effect on the risk of lung infection. If evidence suggested that iron metabolism levels influenced the mediators, subsequently affecting infection risk, we employed the “product of coefficients” method (VanderWeele 2016) to evaluate the indirect effects of iron metabolism levels on infection risk through each potential mediator. Standard errors for the indirect effects were derived using the delta method (Carter et al. 2019).

## 3 Results

### 3.1 Genetic Instruments Selection

Through meta‐analysis of GWAS across three populations, we identified 10 SNPs associated with increased serum iron levels, 28 SNPs associated with increased ferritin levels, 6 SNPs associated with increased TSAT, and 8 SNPs associated with decreased TIBC. Additionally, we identified 2 SNPs associated with decreased serum lactoferrin levels, 7 SNPs associated with increased liver iron content, 3 SNPs associated with decreased liver iron content, 41 SNPs associated with increased neutrophil levels, 48 SNPs associated with increased monocyte levels, 5 SNPs associated with decreased ferritin levels, and 6 SNPs associated with decreased LCN2 levels. From these, we extracted SNP‐exposure correlations. These SNPs (Table S2) were selected for further main analyses.

### 3.2 MR Analysis of Iron Related Biomarkers and Susceptibility to Respiratory Infectious Diseases

#### 3.2.1 MR Analysis of Iron Related Biomarkers and Susceptibility to Bacterial Pneumonia

Increased serum iron levels (OR, 0.85; 95% CI, 0.73–0.99; *p* = 0.04), increased TSAT (OR, 1.32; 95% CI, 1.03–1.70; *p* = 0.03), decreased TIBC (OR, 1.19; 95% CI, 1.05–1.35; *p* = 0.005) and increased liver iron content (OR, 1.11; 95% CI, 1.03–1.19; *p* = 0.007) have associations with susceptibility to bacterial pneumonia.

**FIGURE 2:**
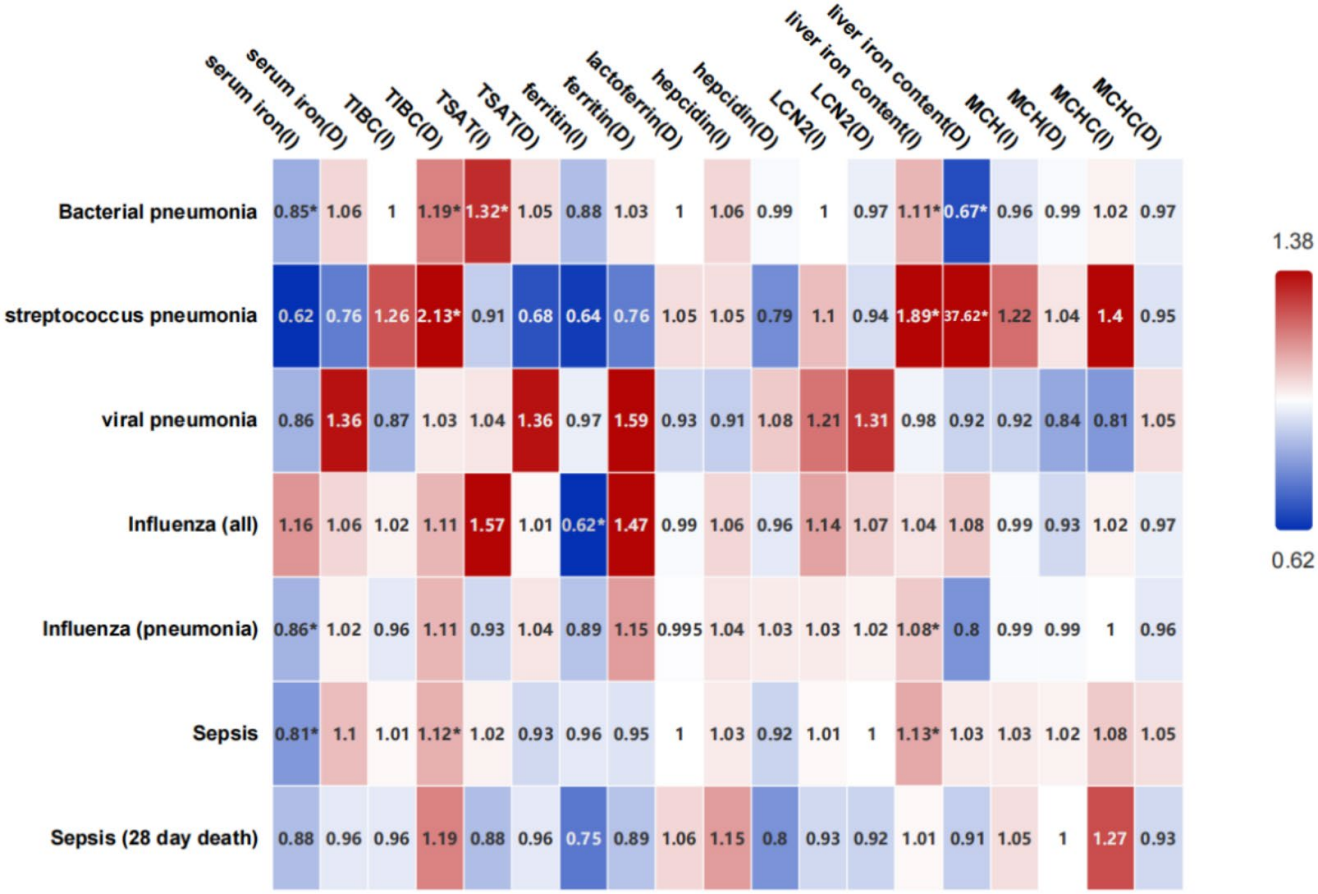
Association between iron related biomarkers and susceptibility to respiratory infectious diseases using inverse variance weighted. The horizontal axis represents exposure, the vertical axis represents outcome, the values in the box are OR values, and “*” represents *p* < 0.05. D, decreased; I, increased; LCN2, neutrophil gelatinase‐associated lipocalin; TIBC, total iron‐binding capacity; TSAT, transferrin saturation.

Liver iron content (increased: OR, 1.11; 95% CI, 1.03–1.19; *p* = 0.007; decreased: OR, 37.62; 95% CI, 35.41–39.83; *p* = 0.0013), decreased TIBC (OR, 2.13; 95% CI, 1.51–2.74; *p* = 0.016) correlated with susceptibility to pneumonia caused by *Streptococcus pneumoniae* (Figure 2).

#### 3.2.2 MR Analysis of Iron Related Biomarkers and Susceptibility to Viral Pneumonia

No iron markers were associated with susceptibility to viral pneumonia. Additionally, we separately analyzed the relationship between iron and influenza. An increased ferritin level was associated with susceptibility to influenza (OR, 0.62; 95% CI, 0.24–0.99; *p* = 0.01). Increased serum iron levels (OR, 0.86; 95% CI, 0.73–0.99; *p* = 0.03) and increased liver iron content (OR, 1.08; 95% CI, 1.01–1.15; *p* = 0.03) have associations with susceptibility to influenza pneumonia (Figure 2).

#### 3.2.3 Iron Related Biomarkers and Susceptibility to Sepsis MR Analysis

Increased serum iron levels (OR, 0.81; 95% CI, 0.72–0.91; *p* = 0.0004), increased liver iron contents (OR, 1.13; 95% CI, 1.05–1.21; *p* = 0.0007) and decreased TIBC (OR, 1.12; 95% CI, 1.04–1.21; *p* = 0.005) have an association with susceptibility to sepsis. No iron marker was associated with the risk of 28‐day mortality in sepsis (Figure 2).

### 3.3 MR Analysis of the Correlation Between Iron Related Biomarkers and Immune Cells

**FIGURE 3:**
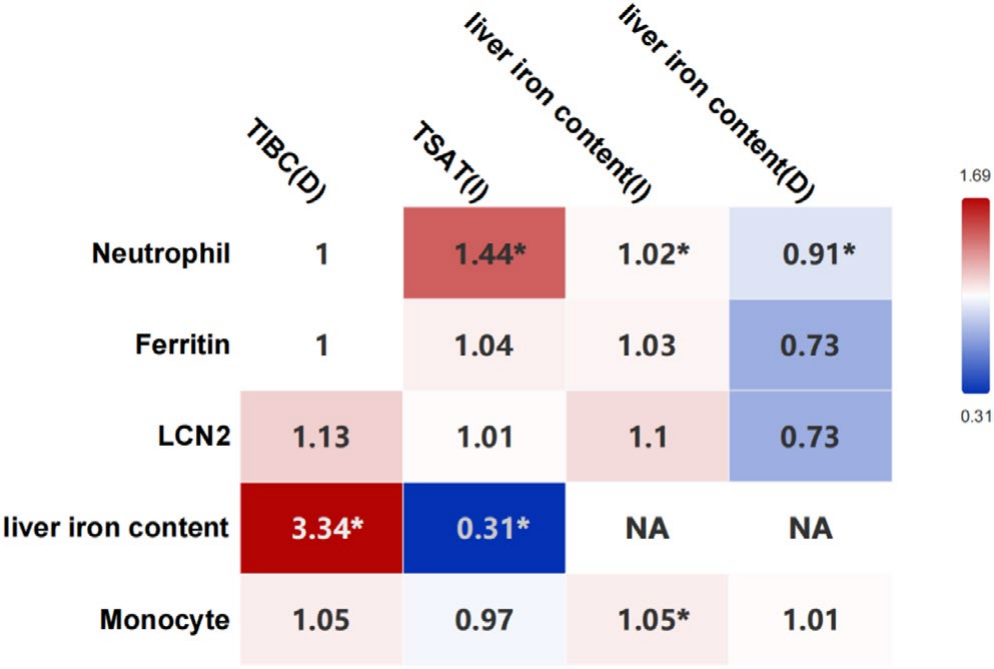
Association between iron related biomarkers and inflammation and immune cells using inverse variance weighted. The horizontal axis represents exposure, the vertical axis represents outcome, the values in the box are OR values, and “*” represents *p* < 0.05. D, decreased; I, increased; LCN2, neutrophil gelatinase‐associated lipocalin; TIBC, total iron‐binding capacity; TSAT, transferrin saturation.

Serum iron level (increased: OR, 1.08; 95% CI, 1.00–1.16; *p* = 0.04; decreased: OR, 0.96; 95% CI, 0.94–0.97; *p* < 0.00001), ferritin level (increased: OR, 0.88; 95% CI, 0.85–0.92; *p* < 0.00001; decreased: OR, 0.91; 95% CI, 0.85–0.97; *p* = 0.005), TSAT (increased: OR, 1.44; 95% CI, 1.30–1.60; *p* < 0.00001; decreased: OR, 0.96; 95% CI, 0.95–0.98; *p* < 0.00001), decreased lactoferrin levels (OR, 0.99; 95% CI, 0.99–1.00; *p* = 0.02), liver iron content (increased: OR, 1.02; 95% CI, 1.01–1.03; *p* = 0.0004; decreased: OR, 0.91; 95% CI, 0.87–0.95; *p* < 0.0001) correlated with neutrophil levels (Figure 3). Decreased ferritin level (OR, 0.95; 95% CI, 0.92–0.98; *p* = 0.003), increased liver iron content (OR, 1.05; 95% CI, 1.04–1.07; *p* < 0.00001) correlated with monocyte levels (Figure 3). A leave‐one‐out analysis was performed and the above results remained robust.

### 3.4 MR Analysis of Immune Cells and Susceptibility to Respiratory Infectious Diseases

**FIGURE 4:**
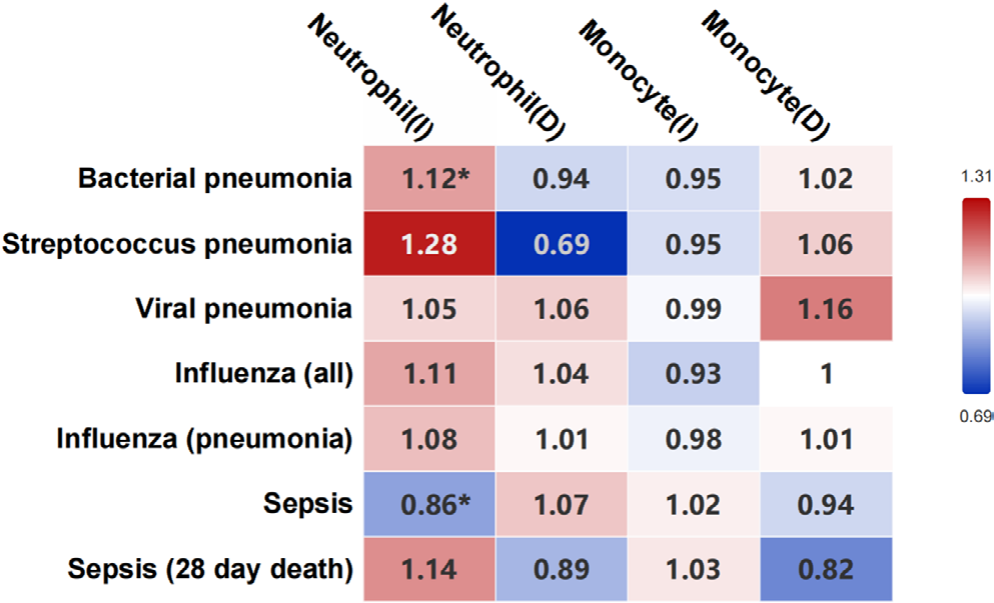
Association between immune cells and susceptibility to respiratory infectious diseases using inverse variance weighted. The horizontal axis represents exposure, the vertical axis represents outcome, the values in the box are OR values, and “*” represents *p* < 0.05. D, decreased; I, increased.

Increased neutrophil count (OR, 1.12; 95% CI, 1.02–1.23; *p* = 0.01) was associated with susceptibility to bacterial pneumonia. An increased neutrophil count (OR, 0.86; 95% CI, 0.78–0.96; *p* = 0.004) was associated with susceptibility to sepsis. There was no statistically significant association between neutrophil count and viral pneumonia, influenza pneumonia, or pneumonia due to *Streptococcus pneumoniae* (Figure 4).

### 3.5 The Interaction Between Iron, Immune Cells, Cytokines, and Diseases

IL‐1β (increased: OR, 1.05; 95% CI, 1.01–1.09; *p* = 0.02; decreased: OR, 0.93; 95% CI, 0.87–0.99; *p* = 0.01), IL‐6 (increased: OR, 0.95; 95% CI, 0.93–0.97; *p* < 0.00001; decreased: OR, 0.96; 95% CI, 0.94–0.99; *p* = 0.001), decreased MCH (OR, 0.93; 95% CI, 0.92–0.95; *p* < 0.00001), Monocyte count (increased: OR, 1.14; 95% CI, 1.11–1.17; *p* < 0.00001; decreased: OR, 1.22; 95% CI, 1.18–1.26; *p* < 0.00001) were associated with neutrophil levels.

Increased LCN2 (OR, 1.04; 95% CI, 1.01–1.07; *p* = 0.01), increased IL‐6 (OR, 0.97; 95% CI, 0.95–0.98; *p* < 0.0001), MCH (increased: OR, 0.97; 95% CI, 0.95–0.99; *p* = 0.0004; decreased: OR, 0.96; 95% CI, 0.94–0.98; *p* < 0.0001), decreased MCHC (OR, 0.94; 95% CI, 0.88–1.00; *p* = 0.05), and neutrophil count (increased: OR, 1.22; 95% CI, 1.18–1.26; *p* < 0.00001; decreased: OR, 1.42; 95% CI, 1.36–1.49; *p* < 0.00001) were associated with monocyte levels.

Serum iron level (increased: OR, 0.22; 95% CI, −0.39–0.83; *p* = 1.1e‐06; decreased: OR, 0.61; 95% CI, 0.47–0.74; *p* = 1.44e‐12), TSAT (increased: OR, 0.31; 95% CI, 0.06–0.56; *p* = 1.7e‐20; decreased: OR, 0.66; 95% CI, 0.46–0.85; *p* = 2.26e‐05), decreased TIBC (OR, 3.34; 95% CI, 3.14–3.53; *p* = 1.22e‐33), and increased LCN2 (OR, 1.08; 95% CI, 1.02–1.14; *p* = 0.01) correlated with liver iron content. Increased ferritin level is associated with decreased hepcidin level.

**FIGURE 5:**
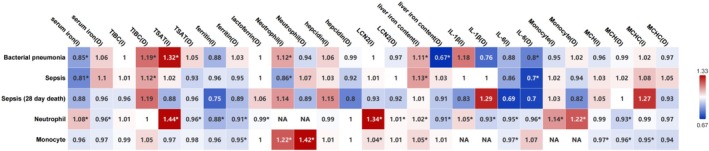
The interaction between iron, immune cells, cytokines, and diseases using inverse variance weighted. The horizontal axis represents exposure, the vertical axis represents outcome, the values in the box are OR values, and “*” represents *p* < 0.05. D, decreased; I, increased; LCN2, neutrophil gelatinase‐associated lipocalin; TIBC, total iron‐binding capacity; TSAT, transferrin saturation.

There was no association between hepcidin, LCN2, and various infectious diseases. Increased levels of IL‐1β were positively correlated with the susceptibility to pneumococcal pneumonia (OR, 4.12; 95% CI, 2.96–5.28; *p* = 0.017). Decreased IL‐6 levels (OR, 0.8; 95% CI, 0.66–0.97; *p* = 0.02) were associated with susceptibility to bacterial pneumonia. Decreased IL‐6 levels were negatively correlated with susceptibility to sepsis (OR, 0.7; 95% CI, 0.55–0.88; *p* = 0.002) (Figure 5).

### 3.6 Mediation Analysis

Neutrophil acts as an intermediary factor in the susceptibility to bacterial pneumonia by TSAT. We estimated the indirect effect of increased TSAT on bacterial pneumonia via neutrophil and found that the mediation effect of neutrophil was OR = 1.008 (95% CI, 1.001–1.015; *p* = 0.036) with a mediated proportion of 6.5% (95% CI, 5.7%–7.2%). No statistically significant results were obtained for other mediation analysis pathways (Table S5).

### 3.7 Reverse MR Analysis

**FIGURE 6:**
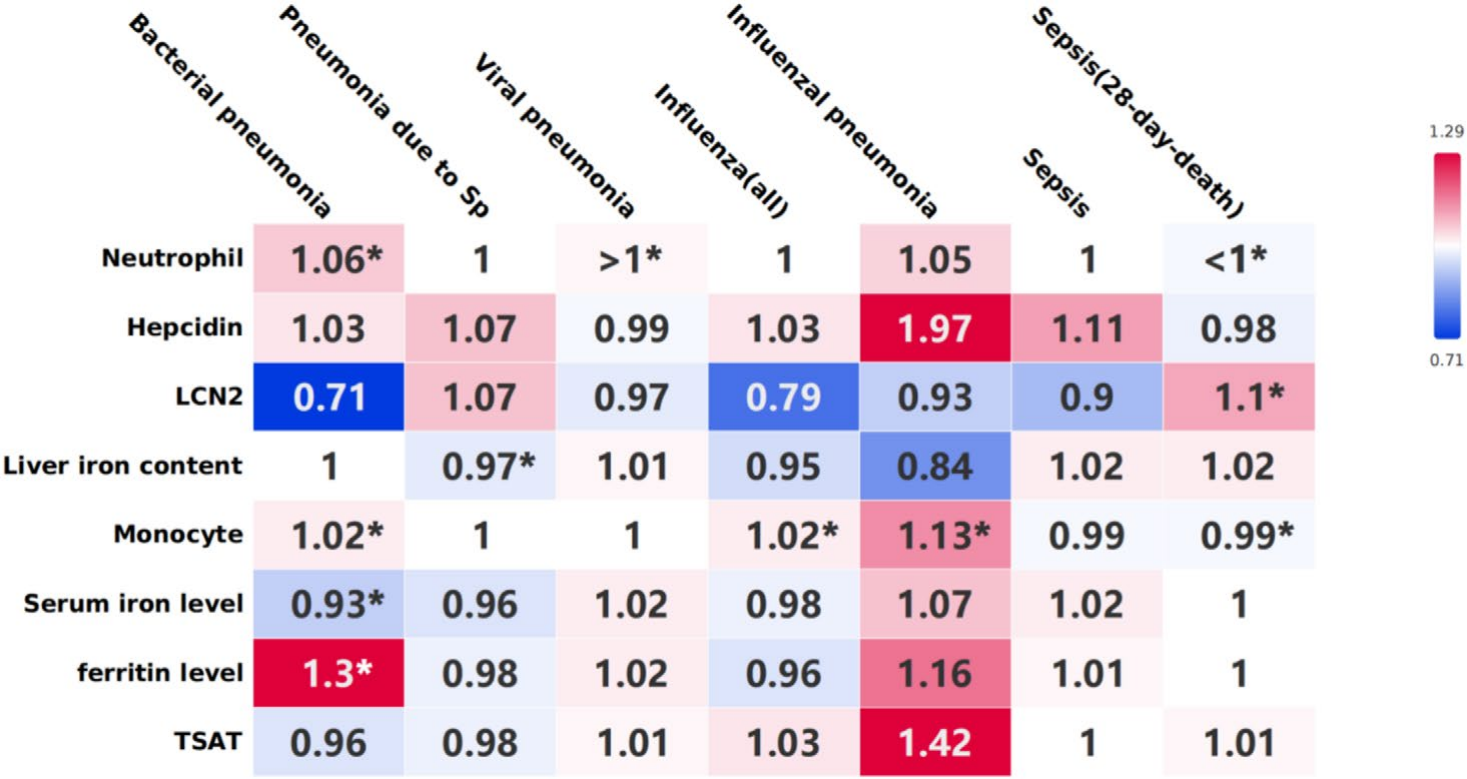
Summary of reverse mendelian randomization analysis results using inverse variance weighted. The horizontal axis represents exposure, the vertical axis represents outcome, the values in the box are OR values, and “*” represents *p* < 0.05. LCN2, neutrophil gelatinase‐associated lipocalin; Sp, *Streptococcus pneumoniae* ; TSAT, transferrin saturation.

In reverse MR analyses, bacterial pneumonia leads to a decrease in serum iron levels, an increase in ferritin levels, neutrophil count, and monocyte count. *Streptococcus pneumoniae* is associated with a decrease in liver iron content. Viral pneumonia results in an increase in neutrophil count, while influenza or influenza‐associated pneumonia leads to an increase in monocyte count. It was not found that sepsis affects the aforementioned iron metabolism markers or changes in neutrophil and monocyte counts. However, the results suggest a possible relationship between decreased neutrophils and monocytes and a 28‐day mortality risk in sepsis (Figure 6).

### 3.8 Sensitivity Analysis

To further assess the potential pleiotropic effects of the genetic instruments used in this study, we conducted MR‐Egger regression and leave‐one‐out analysis. In the MR analysis of iron metabolism levels and infection risk, we found no evidence supporting the presence of unbalanced pleiotropy in the genetic instruments (Figure S2–8). Under the IVW assumption, we assume these SNPs exhibit no pleiotropy. Given that all pleiotropy tests yielded statistically non‐significant results, indicating the absence of pleiotropic effects of the genetic instruments, we relied on IVW estimates as the primary outcome.

## 4 Discussion

Iron is an essential element for all living organisms. Both microorganisms and human beings face challenges in acquiring sufficient iron from the living environment. The iron competition between pathogens and hosts affects the survival strategy of pathogens in the host environment and the defense strategy of the host against microbes (Schaible and Kaufmann 2004). Inflammatory anemia is often observed in patients with infectious diseases in clinical practice, which is considered a response of the host to transfer iron ions from the serum into cells, forming a low serum iron environment to prevent pathogen growth and proliferation—referred to as nutritional immunity (Mantovani and Garlanda 2023). Iron serves as a regulator of both innate and adaptive immunity; whether it is iron deficiency or iron overload significantly impacts the function of the immune system. Ferritin has shown a notable role in monitoring infectious diseases; furthermore, recent studies have suggested that iron metabolism levels may be associated with an increased risk of COVID‐19 or worse prognosis (Mahroum et al. 2022; Chaubey et al. 2023; Tural Onur et al. 2021; Lv et al. 2021). However, few studies have explored the predictive role of iron metabolism levels on the susceptibility to infectious diseases, and how iron metabolism participates in the onset and progression of infections is also unclear (Shah et al. 2021). Therefore, we hypothesize that iron metabolism levels may influence the susceptibility to pneumonia or sepsis. To comprehensively understand the impact of changes in iron metabolism levels on the risk of LRIs and to explore the potential biological pathways that changes in iron metabolism contribute to infection, we conduct an MR study to set iron metabolism indicators as exposure factors, immune cells as mediators, pneumonia and sepsis as outcomes, and innovatively analyze the gene–gene associations through the entire pathophysiological process involving iron metabolism–immune cells–cytokines–diseases. This study indicates that iron metabolism levels could affect the susceptibility to LRIs and worse outcomes by regulating the quantity and function of immune cells.

Our MR findings showed that (1) lower risk of bacterial pneumonia was associated with increased serum iron levels and decreased liver iron content, while higher risk was related to reduced TIBC, increased TSAT, and increased liver iron content. These findings were similar to some previous studies, and the reason may relate to a diet rich in iron which increases hepcidin levels and mediates specific infection resistance (Agoro et al. 2017; Stefanova et al. 2017). Ren and colleagues (2021) found that patients with cerebral infarction had higher serum iron levels; the risk of stroke‐associated pneumonia (SAP) significantly decreased, particularly when serum iron level was ≥ 7.8 μmol/L. The U‐shaped association between abnormal hepatic iron content and the risk of pneumococcal pneumonia may stem from bidirectional regulation of host–pathogen interactions mediated by disrupted iron homeostasis (Drakesmith and Zoller 2024). On one hand, under iron‐deficient conditions (e.g., chronic blood loss, or inflammatory iron restriction), insufficient hepatic iron stores impair immune defenses such as neutrophil extracellular trap (NET) formation and complement activation, while simultaneously forcing the host to release stored iron for erythropoiesis, inadvertently providing pathogens with accessible iron. On the other hand, the host initiates a nutritional immune response, transporting serum iron into liver cells and Kupffer cells. Liver iron content overload may limit bacterial iron accessibility by inducing heme oxygenase‐1 (HO‐1) to degrade free heme, while simultaneously promoting the secretion of iron‐saturated lactoferrin (Yeh et al. 2018; Furuyama et al. 2007). Furthermore, iron‐laden Kupffer cells enhance bacterial clearance via reactive oxygen species (ROS)‐dependent killing and defensin secretion, thereby reducing hematogenous dissemination (Kohgo et al. 2007). However, this mechanism inadvertently provides a chance to *Streptococcus pneumoniae* which relies on the PiuABCD system for heme iron acquisition (Yang et al. 2019). As a host defense mediator, hepcidin increases in response to infection and inflammation, blocking iron delivery through ferroprotein to blood plasma, thus limiting iron availability to invading microbes (Nemeth and Ganz 2021). However, liver iron content overload also leads to *Hfe* (a gene of hemochromatosis protein) downregulation, affecting hepcidin level (Ahmad et al. 2002), which disrupted the above effect and further impaired the neutrophil recruitment and macrophage phagocytosis in the inflammatory response in the lungs responding to inflammation (Comità et al. 2024; Oppen et al. 2021; Benesova et al. 2012). Moreover, it may also be related to the reduced transferrin TSAT levels, weakening the protective iron‐sequestering ability of humans, facilitating bacterial iron pillaging and proliferation (Wu et al. 2022). Recent studies (Nakashima et al. 2022; Wang et al. 2017) have found that *Streptococcus pneumoniae* in the liver could cause abdominal infection, suggesting that hepatic B cells and macrophages might be involved in phagocytosing *Streptococcus pneumoniae* , but disturbances in liver iron content could impair this phagocytic function, leading to hematogenous spread and subsequent pulmonary infections (Benesova et al. 2012; Wang et al. 2017; An et al. 2022). (2) Susceptibility to viral pneumonia was not directly related to iron metabolism levels, but lower risk of influenza pneumonia was associated with higher serum iron levels and higher serum ferritin levels, while higher risk was related to increased liver iron content. This may suggest that different respiratory viruses differ in their ability to interfere with the host's iron metabolism. Recent research indicates that metastable iron sulfide (mFeS) can inhibit ferroptosis, protecting cells from intracellular *influenza virus* (Miao et al. 2023). Some consensus also pointed out that correcting blood iron levels before the influenza season may reduce the incidence of influenza and its complications (Cirovic and Cirovic 2023). In addition, the liver contains a large number of CD8(+) T cells and invariant natural killer T (iNKT) cells that have specific immune effects against the *influenza virus*. Compared to the lungs, the liver has higher numbers of apoptotic CD8(+) T cells, reflecting the severity of influenza pneumonia (Belz et al. 1998). Additionally, influenza virus infection in mice can induce memory‐like iNKT cells that protect mice from secondary influenza virus infections (Humeniuk et al. 2023; Li et al. 2017). Studies have shown that iron‐rich diets could promote hepcidin upregulation and increase T cell recruitment (Drakesmith and Prentice 2008). Both anemia (low serum iron) and overloaded tissue iron (liver iron content) could decrease lymphocyte activity, thereby increasing infection risk, which explains this phenomenon. (3) Low risk of sepsis progression for infectious disease patients is associated with higher serum iron levels, whereas high risk is linked to decreased TIBC and increased liver iron content. However, previous MR studies (Hu et al. 2021; Mohus et al. 2022; Hamilton et al. 2023) and cohort studies (Mohus et al. 2018; Mottelson et al. 2024) found that higher serum iron levels were associated with an increased risk of sepsis, and similarly we found decreased TIBC and increased liver iron content, both indicating elevated serum iron levels may increase sepsis susceptibility, and paradoxically we found elevated serum iron may reduce sepsis susceptibility. This discrepancy highlights the apparently different conclusions regarding the role of serum iron levels in sepsis risk. However, this set of contradictory results is highly intriguing and can also be readily explained: (a) Overloaded liver iron content, as previously mentioned, impairs immune cell function, increases infection risk, leads to ROS increase and GSH reduction, exacerbates liver cell damage, and then increases sepsis susceptibility (Jung et al. 2000). Studies have shown that lower serum iron levels are associated with higher ICU admission rates and higher treatment failure rates in hospital‐acquired pneumonia patients. Increased serum iron levels could promote hepcidin expression in the liver (Stoffel et al. 2017; Moretti et al. 2015); high hepcidin expression has a negative feedback effect on serum iron to achieve iron homeostasis (Billesbølle et al. 2020). High serum iron levels or hepcidin do not affect other key components of innate immunity or promote intracellular and certain extracellular infections, and hepcidin analogs might help treat iron‐dependent infections (Stefanova et al. 2017). Hepcidin could inhibit bacterial growth and protect organs, thus blocking the progression of infection to sepsis (Prentice et al. 2019; Scindia et al. 2019; Zeng et al. 2015; Stefanova et al. 2018). (b) Our data integrated with previous studies to properly demonstrate that all excessively high and low iron metabolism levels in the host are detrimental, posing risks at both ends of iron status, and a dynamic balance should be maintained for health. In other words, there exists a U‐shaped risk curve of iron status and susceptibility to infections and sepsis. Of course, this is also because MR analysis cannot be fully used to study nonlinear correlations, which is an inherent limitation of MR research.

Neutrophils are crucial immune cells for combating infections, and their quantity and function are influenced by iron levels. The activity of myeloperoxidase in neutrophils from iron‐deficient rats was significantly reduced (Mackler et al. 1984). Neutrophils (PMNs) from patients with secondary iron overload had increased iron and ferritin content, as well as defects in phagocytosis (Cantinieaux et al. 1999). Therefore, as we have found in this study, whether iron deficiency (decreased serum iron level) or overload (increased intracellular iron content) could impair the number of neutrophils and potentially affect its ability to defend against the invasion of pathogens. Further analysis in mediation effect innovatively found elevated TSAT could regulate neutrophils increased, leading to higher susceptibility to bacterial pneumonia. TSAT is calculated as serum iron levels divided by TIBC, that is, the proportion of transferrin bound to ferric iron in the serum. On one hand, with stable serum iron levels, the loss of TSAT, reduced TIBC, and increased TSAT, alongside the critical factor of transferrin deficiency in iron competition with bacteria, may be an independent risk factor for susceptibility to respiratory bacterial infections. This may be related to the fact that cytokines produced during the inflammatory response, such as IL‐1, IL‐6, TNF‐α and IFN‐γ, could affect the synthesis of transferrin. It may also be related to the regulation caused by liver iron content overload, resulting from Hepcidin (Bartnikas and Fleming 2012; Rishi et al. 2015; Nemeth et al. 2004). On the other hand, when ferric iron ions in serum bind to transferrin (resulting in increased TSAT) or transfer into cells (resulting in increased liver iron), non‐transferrin‐bound iron (serum free iron) level remains low, and then colonizing bacteria in the tissue may activate the siderophore system to take iron from the serum (Wu et al. 2022). The released siderophores not only directly acquire iron from transferrin to promote bacterial growth and reproduction but also cause systematic inflammation and tissue damage due to their toxicity (Palmer and Skaar 2016). In addition, further analysis the effect on cytokine side in this study revealed that IL‐1β, IL‐6, and LCN2 indeed participated in the mutual regulation of neutrophils, which provides further evidences to support similar conclusions (Karmakar et al. 2020; Allen et al. 2022; Borregaard et al. 2007).

In order to define the impact of bacterial infection on the systematic iron metabolism levels, we conduct a reverse causal analysis in bidirectional MR. In bacterial pneumonia, neutrophils, monocytes, and ferritin were increased, serum iron level was decreased, while liver iron content was reduced in *Streptococcus pneumoniae* pneumonia. Viral infections in the respiratory system led to elevated neutrophils and monocytes but did not cause iron metabolism disorders. However, a decrease in neutrophils and macrophages, along with elevated serum LCN2, predicts a potentially higher 28‐day mortality in sepsis patients. A time‐course clinical study (Moro et al. 2023) found a decrease in serum iron levels after acute bloodstream infection. The change of LCN2 and ferritin has been widely reported in sepsis patients and has become a key indicator for predicting 28‐day mortality in patients with CAP or sepsis (de la Fuente et al. 2024; Zhao et al. 2024; Zhang et al. 2024). Notably, multiple single‐cell transcriptomic analyses have consistently identified neutrophils as the predominant antimicrobial effector cells in murine models of *Escherichia coli* and *Klebsiella pneumoniae* infections (Xie et al. 2020; Cui et al. 2025; Xiao et al. 2025). In the study of mice with a low blood iron phenotype, it was found that neutrophils may be more sensitive to changes in blood iron concentration. Low blood iron can inhibit the production of chemokines and inflammatory factors and impair neutrophil function (Frost et al. 2022; Bonadonna et al. 2022). It suggests that neutrophils are the most crucial immune effector cells, whether in susceptibility before infection or in response after infection. During this process, the disorder occurring on the systematic level of iron metabolism mediated by immunocytes plays key roles in the development and progression of respiratory infectious diseases.

The significance of this study lies in elucidating the clinical phenomenon of increased susceptibility to respiratory infectious diseases in populations with iron metabolism disorders. By linking various dimensions such as iron metabolism–immune cells–cytokines–susceptibility to diseases and prognosis, this study analyzes the genetic correlations in the occurrence and progression of respiratory infectious diseases. It clarifies the connection between iron metabolism disorders and increased susceptibility to bacterial pneumonia, viral pneumonia, and sepsis, highlighting the complex interplay between iron homeostasis and immune defense mechanisms. This provides insights and hints into further research of potential mechanisms. Additionally, the study emphasizes the urgent need for iron metabolism markers to predict and monitor high‐risk groups for respiratory infectious diseases, suggesting that early detection and intervention based on these markers could significantly reduce the risk and adverse prognosis of such diseases.

Literally, this study utilized existing data from large‐scale research and employed genetic variations to investigate the impact of changes in iron metabolism indicators. This approach circumvents the time and resource constraints associated with RCTs and overcomes potential confounding factors and reverse causation limitations inherent in standard observational methods. The inherent limitations of this study can be attributed, to some extent, to the imperfections of the research methodology itself. Firstly, compensatory processes or feedback mechanisms might dilute genetic effects (Lawlor et al. 2008). Although such compensation may mitigate genetic effects, it does not account for the specific associations between genetic proxies of iron metabolism and the risk of infection or sepsis. Secondly, MR analysis assesses lifelong effects, so the magnitude of these effects may not be comparable to short‐term fluctuations in iron levels. Therefore, this study is more meaningful for evaluating the direction of associations rather than providing estimates of effect sizes. In addition, as mentioned earlier, the MR framework is suboptimal for examining U‐shaped associations or nonlinear dose–response relationships between iron homeostasis and infection susceptibility, a methodological constraint arising from its reliance on linear instrumental variable assumptions. This inherent limitation underscores the necessity of complementary approaches (e.g., nonlinear MR extensions or observational cohort analyses) to fully characterize iron status's dual‐risk paradox. However, this MR analysis is valuable for estimating the long‐term impacts of changes in iron metabolism, surpassing the capabilities of RCTs. The reliance on data from European ancestry may constrain the generalizability of our findings to other ethnic populations, especially considering documented genetic heterogeneity in iron metabolism across ancestries. Future validation through multi‐ancestry GWAS is warranted to explore potential ancestry‐specific effects. Additionally, the lack of an external dataset for validation is another limitation of this study.

Considering these potential issues, we have some suggestions on clinical translation in the future: (1) it is also essential for future researchers to further develop relevant population cohorts for observational studies as a necessary; (2) integrating multiple iron metabolism indicators to predict the susceptibility and severity of respiratory infectious diseases, rather than a single iron indicator; (3) according to multi‐Omics (scRNA‐seq, ect.) datasets to verify the correlation between iron‐related SNPs and neutrophil activation markers.

Our MR study provides strong evidence linking variations in iron metabolism indicators to an increased risk of pneumonia and sepsis, while underscoring the need for further research to elucidate the underlying mechanisms. Given that iron level testing has only recently been included in relevant guidelines but is not yet widely practiced clinically, we advocate for clinicians to further construct a cohort of patients with lower respiratory tract infections and sepsis, clarifying the roles of iron metabolism indicators and neutrophils, and consider the importance of monitoring iron levels during clinical assessments to ensure appropriate interventions for patients, which represents a valuable clinical practice strategy. The findings of this study offer high‐quality evidence for disease prevention and control in high‐risk populations globally, particularly for the European population.

## 5 Conclusion

Overall, this MR study confirms that disruptions in iron metabolism increase susceptibility to bacterial pneumonia, viral pneumonia, and sepsis. The U‐shaped risk curve of iron status, shaped by host nutritional immunity mechanisms, demonstrates that both iron deficiency and overload significantly elevate susceptibility to respiratory infections and sepsis, reflecting a biphasic relationship between iron homeostasis and infection risk. Specifically, deficient serum iron level (decreased serum iron levels), excessive serum iron levels (reduced TIBC, increased TSAT), and overload of intracellular iron content (elevated liver iron content) contribute to this phenomenon by modulating neutrophil function and cytokine levels, thereby exacerbating susceptibility to and severity of pneumonia and sepsis. These findings highlight the critical importance of monitoring iron metabolism indicators in high‐risk infection populations in clinical practice and furnish potential insights for mechanistic research and clinical intervention.

## Author Contributions

**Zhenchao Wu:** conceptualization (equal), formal analysis (equal), investigation (equal), methodology (equal), writing – original draft (equal). **Taikang Yao:** data curation (equal), formal analysis (equal), investigation (equal), methodology (equal), writing – original draft (equal). **Zhongyu Han:** formal analysis (equal), writing – original draft (equal). **Zilu Wang:** formal analysis (equal), investigation (equal), writing – original draft (equal). **Beibei Liu:** formal analysis (equal), investigation (equal), writing – review and editing (equal). **Ming Lu:** formal analysis (equal), methodology (equal), writing – review and editing (equal). **Jiajia Zheng:** formal analysis (equal), methodology (equal), writing – review and editing (equal). **Ning Shen:** formal analysis (equal), funding acquisition (equal), methodology (equal), writing – review and editing (equal).

## Ethics Statement

The authors have nothing to report.

## Consent

The authors have nothing to report.

## Conflicts of Interest

The authors declare no conflicts of interest.

## Supporting information


Data S1.

Data S2.

Data S3.

## Data Availability

The original data presented in the study are included in the article/Supporting Information; further inquiries can be directed to the corresponding author.
