# Supplementary material for: Genetic Susceptibility to Causal Relationship Between Iron Metabolism Disorder Involving Immunocytes and Risk of Pneumonia and Sepsis
Source: Food Sci Nutr. 2025 Jun 10;13(6):e70422. doi: 10.1002/fsn3.70422 (PMC12152259; doi:10.1002/fsn3.70422)
Supplement: Supplementary file 1 — Data S1. [file FSN3-13-e70422-s002.docx]

**Supplementary figures**


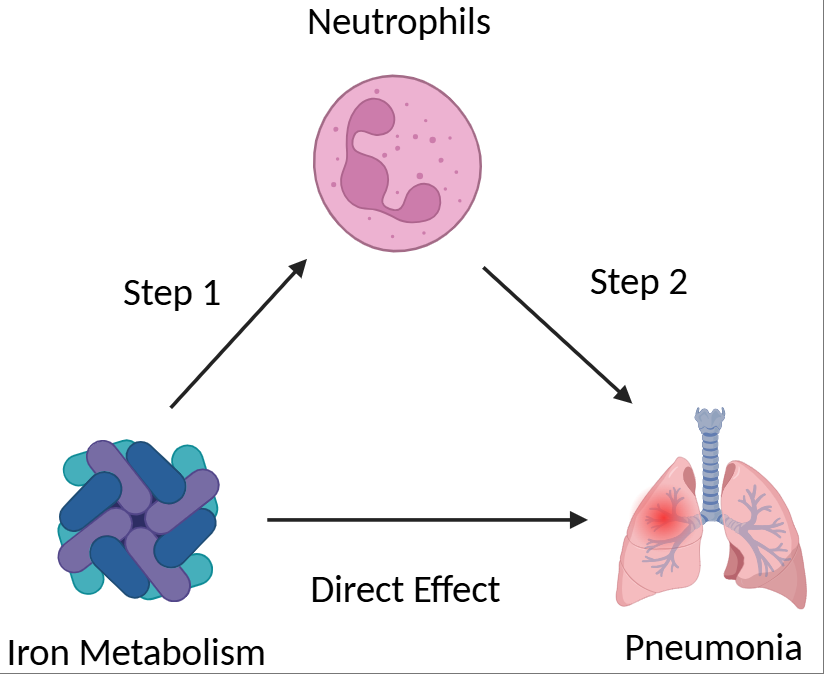


eFig 1. Conceptual framework for the mediation analysis of the relationship between iron metabolism disorders and susceptibility to pneumonia.


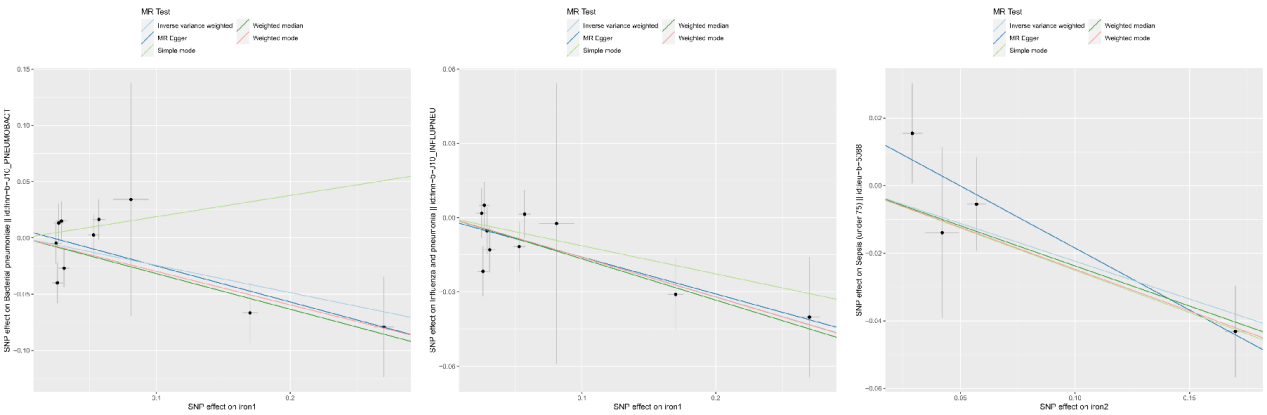


eFig 2. Scatterplot of SNP-specific effects for the associations with iron levels and bacterial pneumonia (left, increased iron levels), influenza pneumonia (middle, increased iron levels), sepsis (under 75) (right, decreased iron levels) for iron levels-associated SNPs, which distinctly shows the respective intercept stem from the IVW, Weighted median and MR Egger, demonstrating the statistically insignificant of the data-driven Egger regression and the subtlety of average pleiotropic effect of each type of infection.


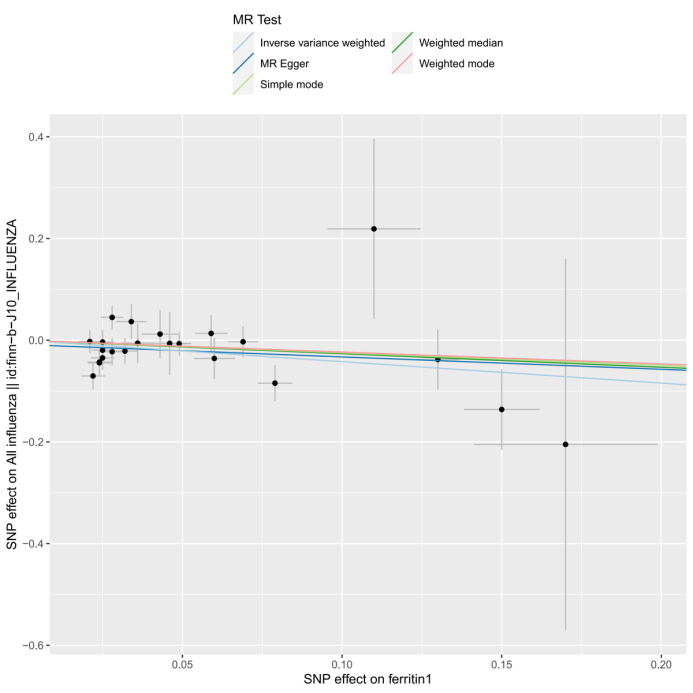


eFig 3. Scatterplot of SNP-specific effects for the associations with increased ferritin levels and influenza for ferritin levels-associated SNPs, which distinctly shows the respective intercept stem from the IVW, Weighted median and MR Egger, demonstrating the statistically insignificant of the data-driven Egger regression and the subtlety of average pleiotropic effect of each type of infection.


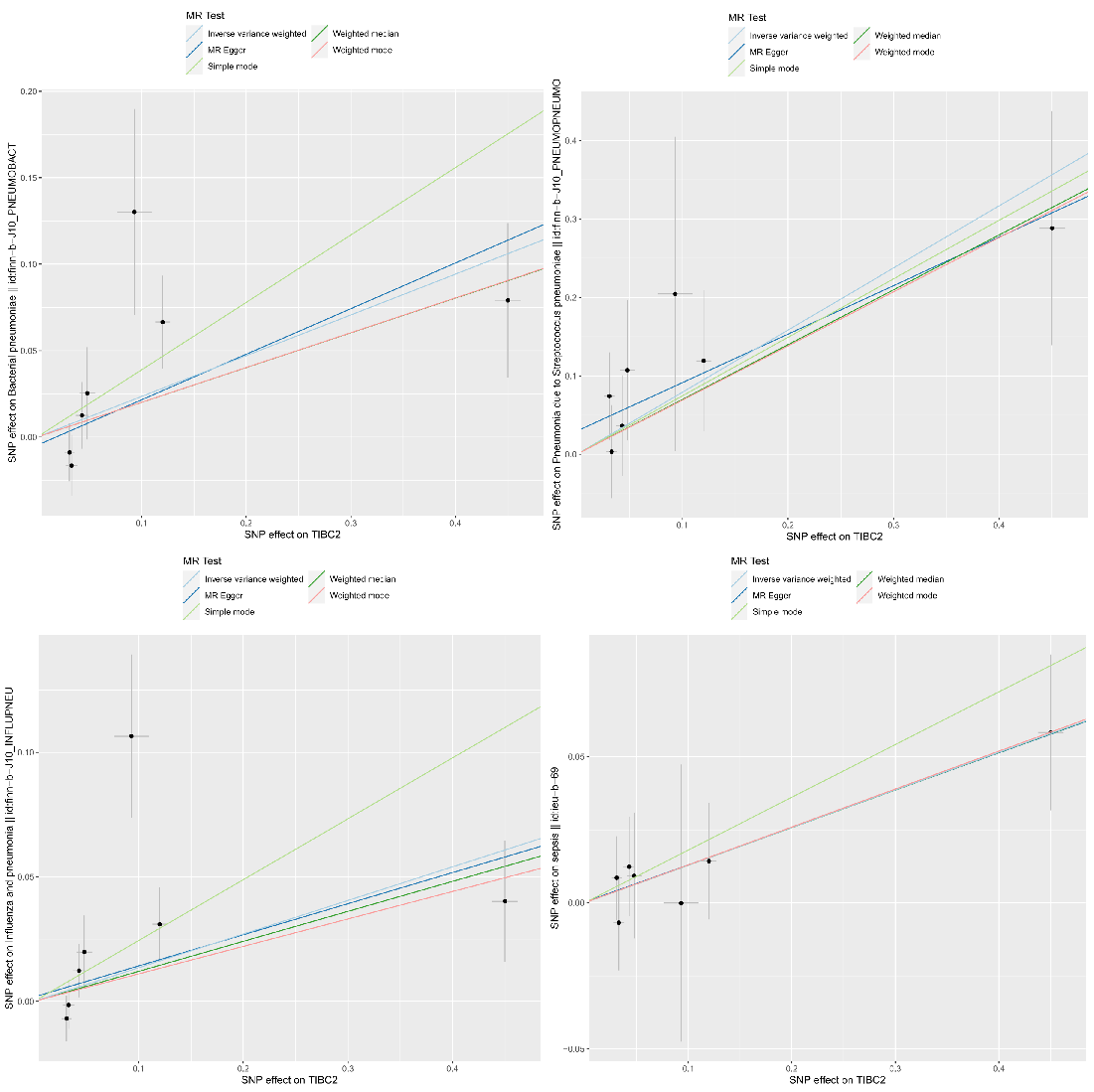


eFig 4. Scatterplot of SNP-specific effects for the associations with decreased TIBC and bacterial pneumonia (top left), pneumonia due to Streptococcus pneumoniae (top right), influenza pneumonia (bottom left), sepsis (bottom right) for TIBC-associated SNPs, which distinctly shows the respective intercept stem from the IVW, Weighted median and MR Egger, demonstrating the statistically insignificant of the data-driven Egger regression and the subtlety of average pleiotropic effect of each type of infection.


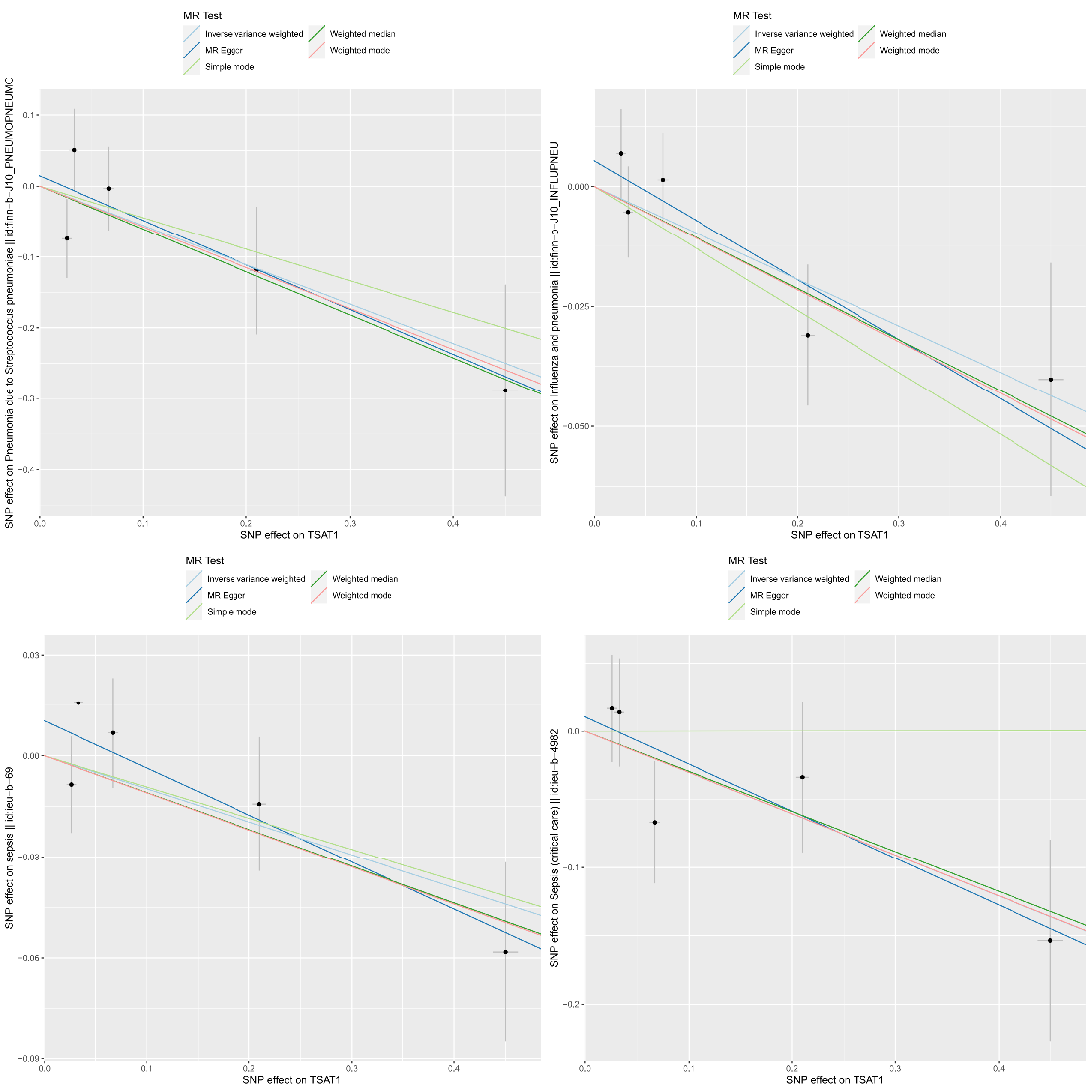


eFig 5. Scatterplot of SNP-specific effects for the associations with increased TSAT and pneumonia due to Streptococcus pneumoniae (top left), influenza pneumonia (top right), sepsis (bottom left), sepsis (28 day death) (bottom right) for TSAT-associated SNPs, which distinctly shows the respective intercept stem from the IVW, Weighted median and MR Egger, demonstrating the statistically insignificant of the data-driven Egger regression and the subtlety of average pleiotropic effect of each type of infection.


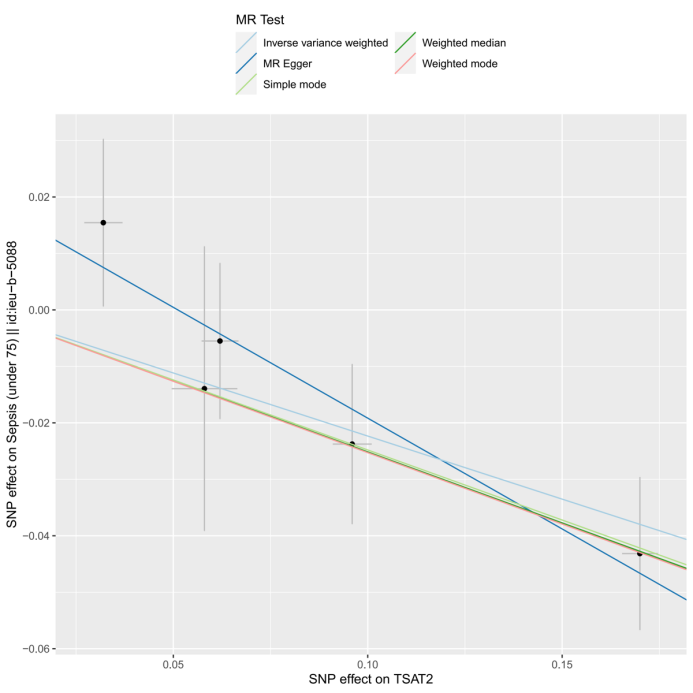


eFig 6. Scatterplot of SNP-specific effects for the associations with decreased TSAT and sepsis (under 75) for TSAT-associated SNPs, which distinctly shows the respective intercept stem from the IVW, Weighted median and MR Egger, demonstrating the statistically insignificant of the data-driven Egger regression and the subtlety of average pleiotropic effect of each type of infection.


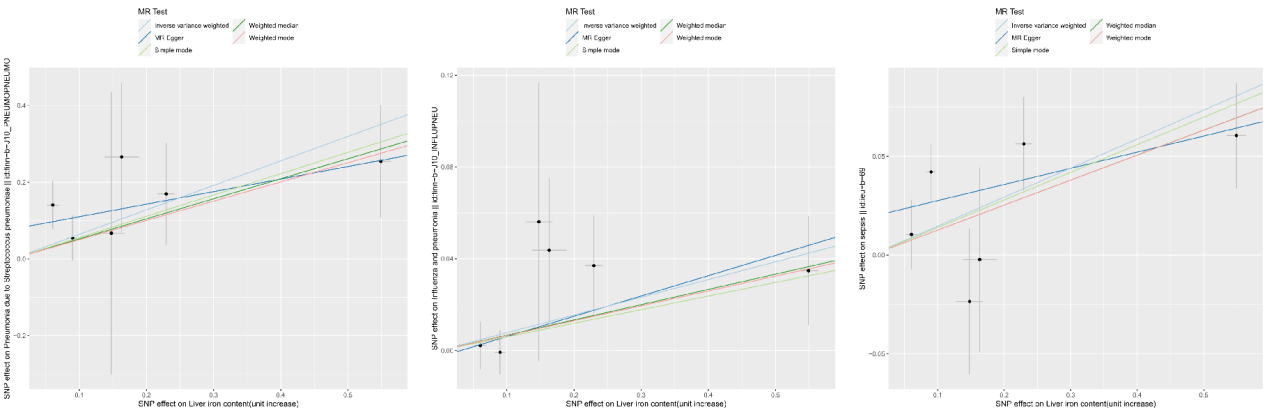


eFig 7. Scatterplot of SNP-specific effects for the associations with increased liver iron content and pneumonia due to Streptococcus pneumoniae (left), influenza pneumonia (middle), sepsis (right) for liver iron content-associated SNPs, which distinctly shows the respective intercept stem from the IVW, Weighted median and MR Egger, demonstrating the statistically insignificant of the data-driven Egger regression and the subtlety of average pleiotropic effect of each type of infection.


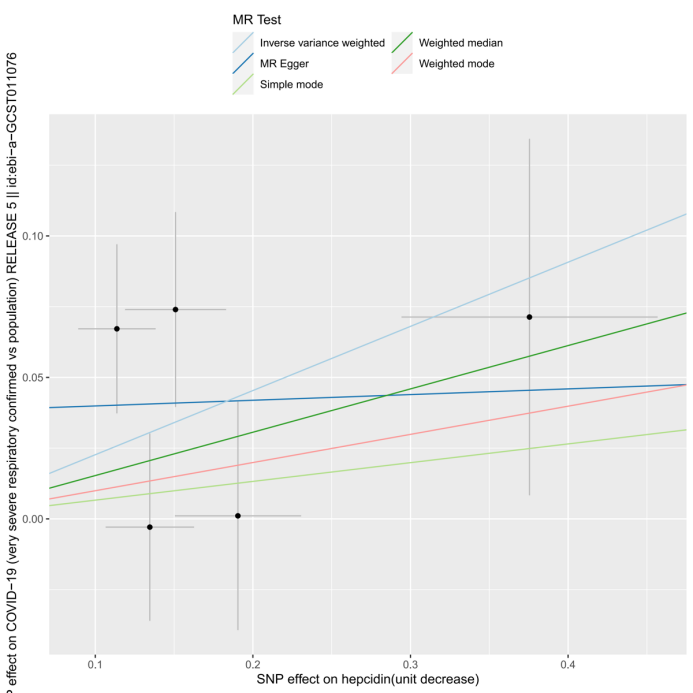


eFig 8. Scatterplot of SNP-specific effects for the associations with decreased hepcidin and COVID-19 (very severe respiratory confirmed vs population) for hepcidin-associated SNPs, which distinctly shows the respective intercept stem from the IVW, Weighted median and MR Egger, demonstrating the statistically insignificant of the data-driven Egger regression and the subtlety of average pleiotropic effect of each type of infection.


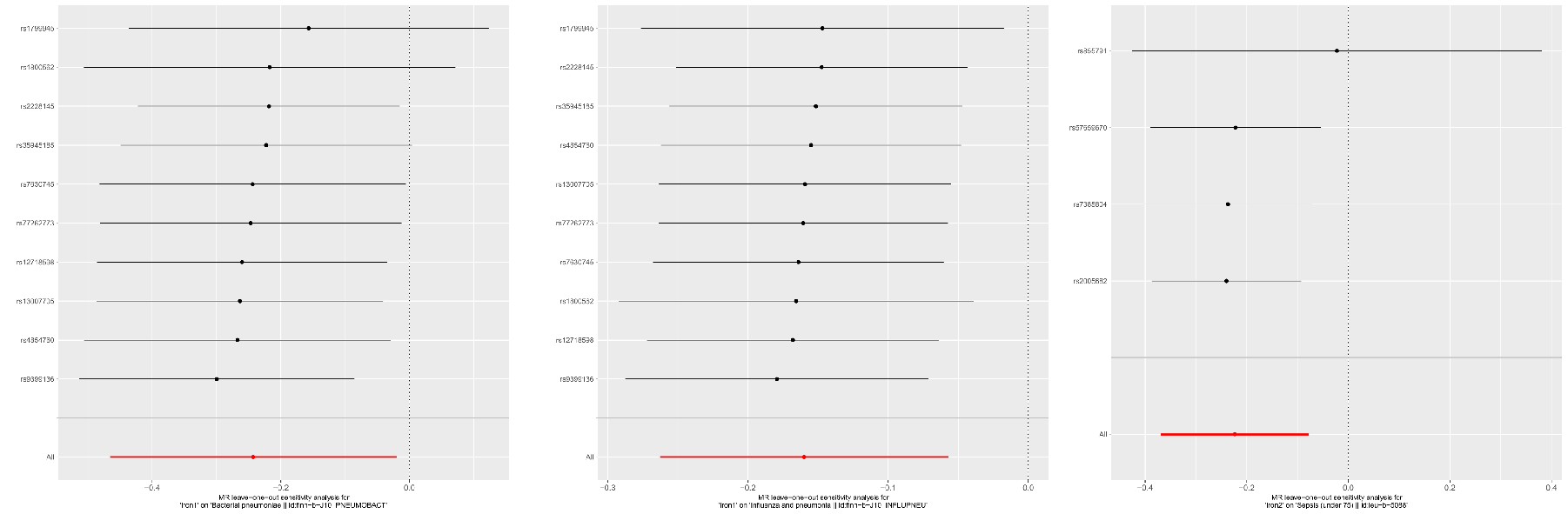


eFig 9. Leave-one-out plots of SNP-specific effects for the associations with iron levels and bacterial pneumonia (left, increased iron levels), influenza pneumonia (middle, increased iron levels), sepsis (under 75) (right, decreased iron levels) .


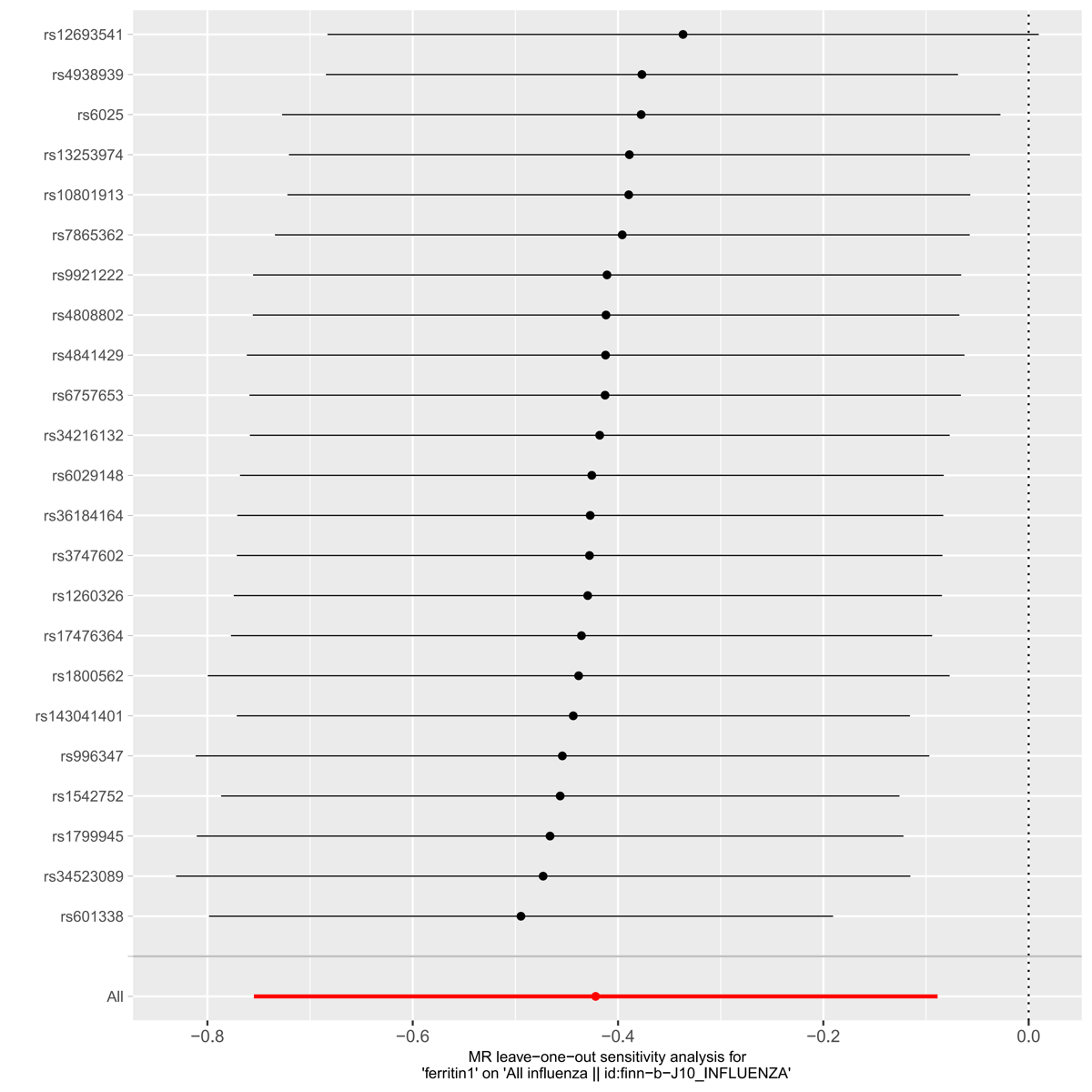


eFig 10. Leave-one-out plots of SNP-specific effects for the associations with increased ferritin levels and influenza.


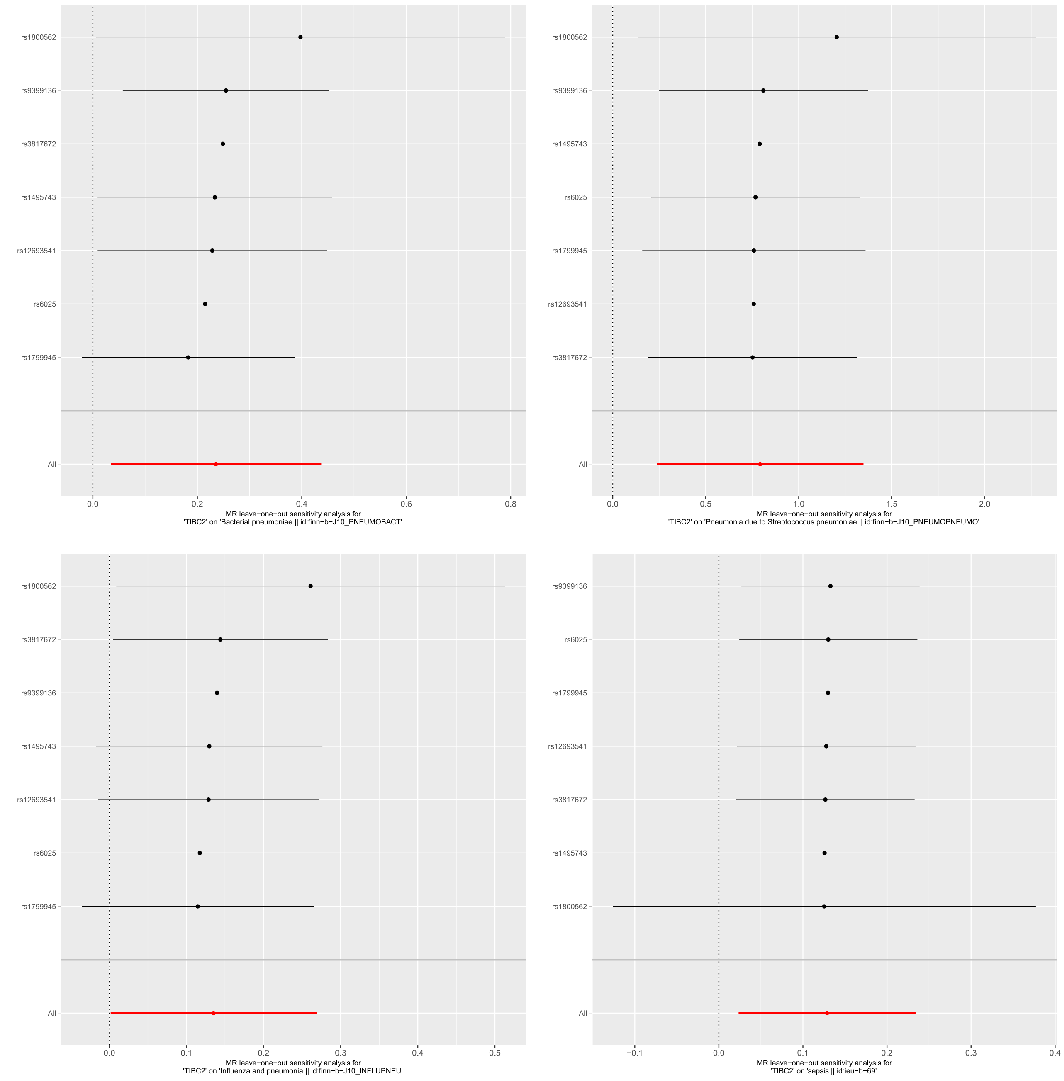


eFig 11. Leave-one-out plots of SNP-specific effects for the associations with decreased TIBC and bacterial pneumonia (top left), pneumonia due to Streptococcus pneumoniae (top right), influenza pneumonia (bottom left), sepsis (bottom right) .


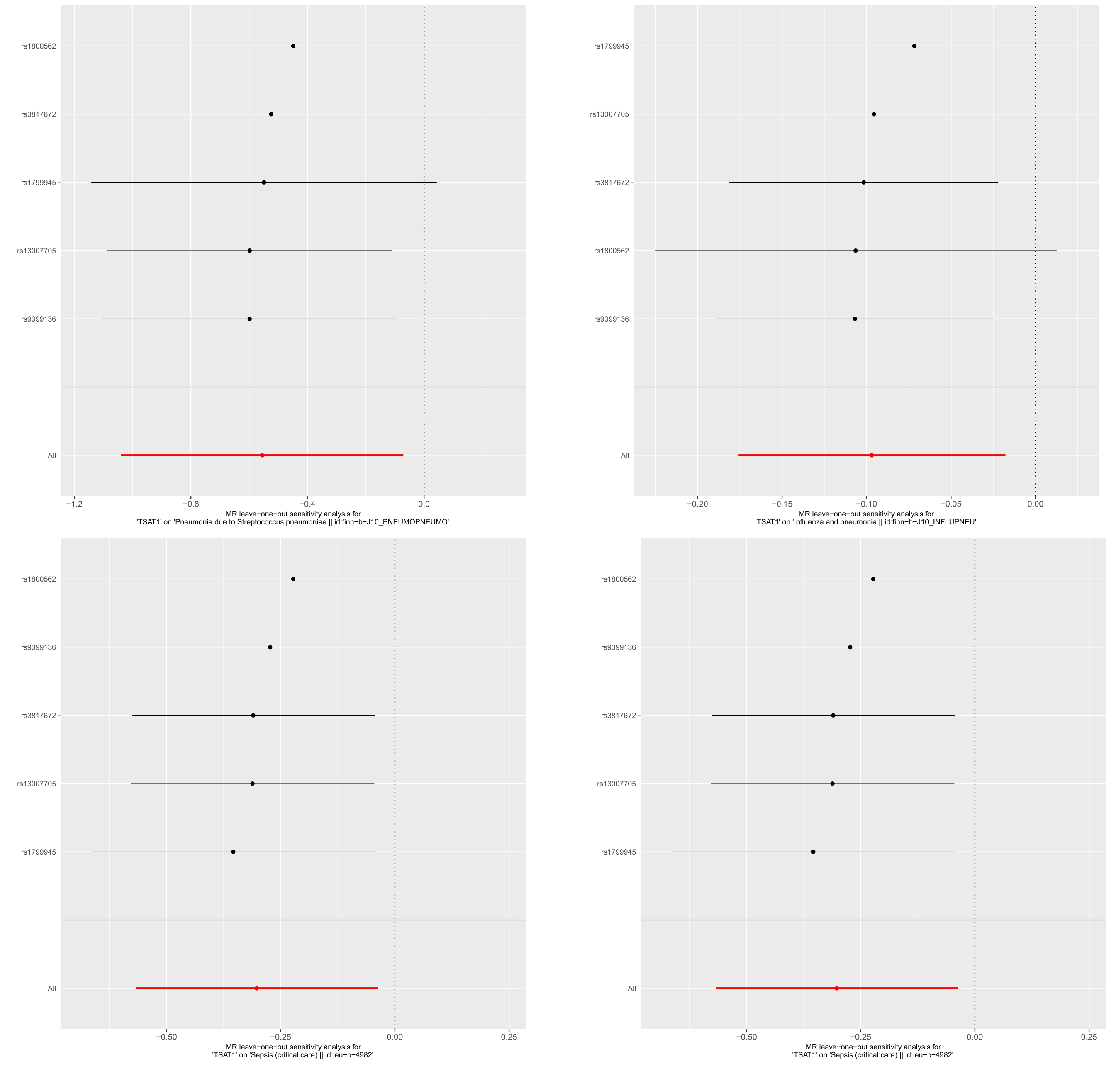


eFig 12. Leave-one-out plots of SNP-specific effects for the associations with increased TSAT and pneumonia due to Streptococcus pneumoniae (top left), influenza pneumonia (top right), sepsis (bottom left), sepsis (28 day death) (bottom right) for TSAT-associated SNPs.


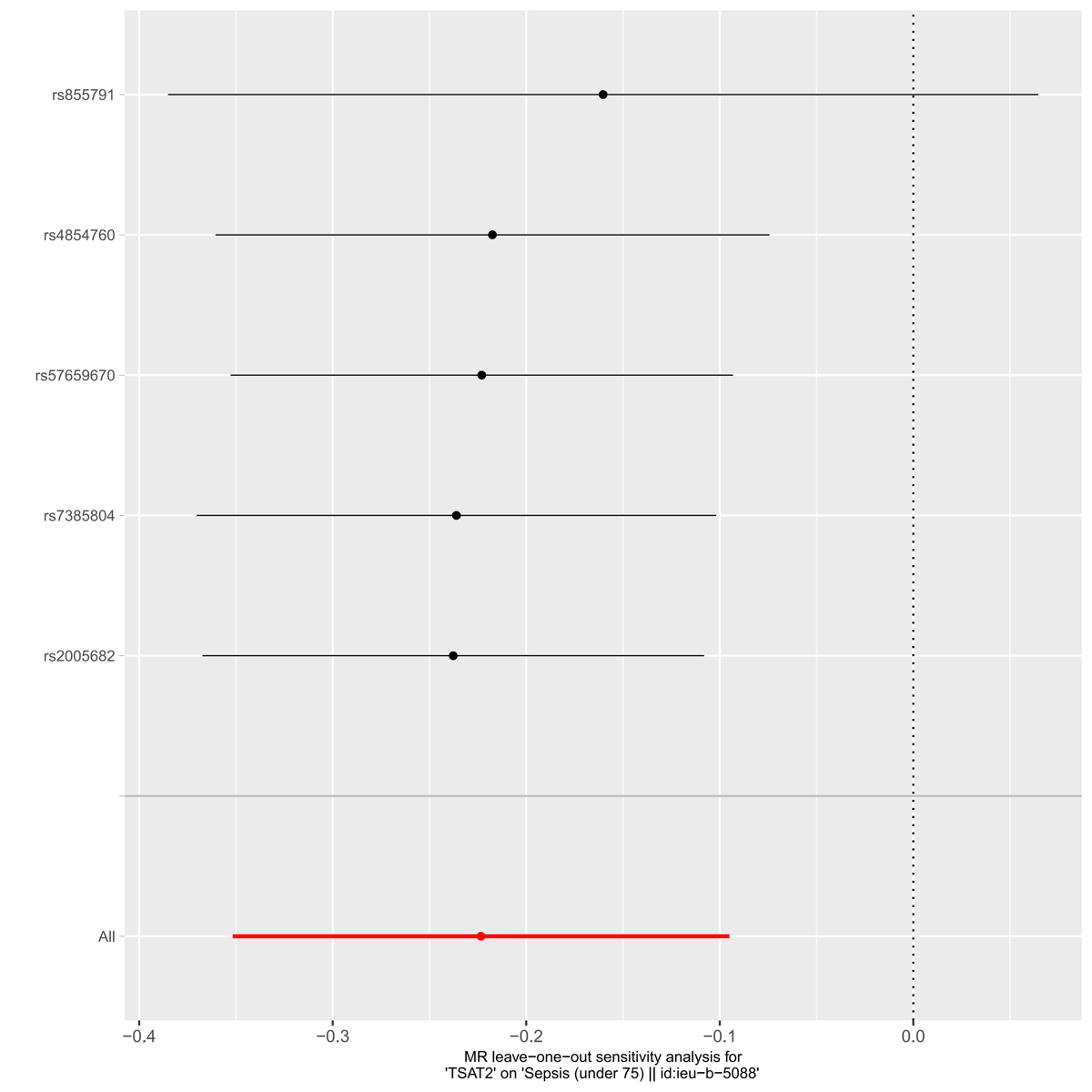


eFig 13. Leave-one-out plots of SNP-specific effects for the associations with decreased TSAT and sepsis (under 75) .


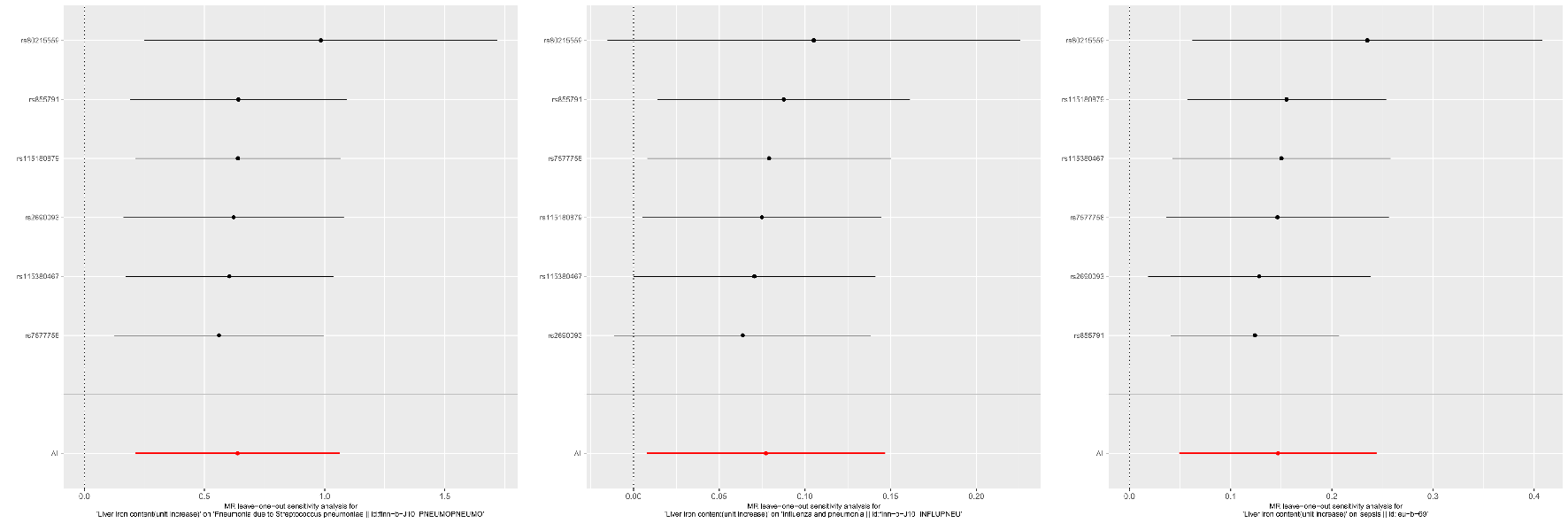


eFig 14. Leave-one-out plots of SNP-specific effects for the associations with increased liver iron content and pneumonia due to Streptococcus pneumoniae (left), influenza pneumonia (middle), sepsis (right) for liver iron content-associated SNPs.


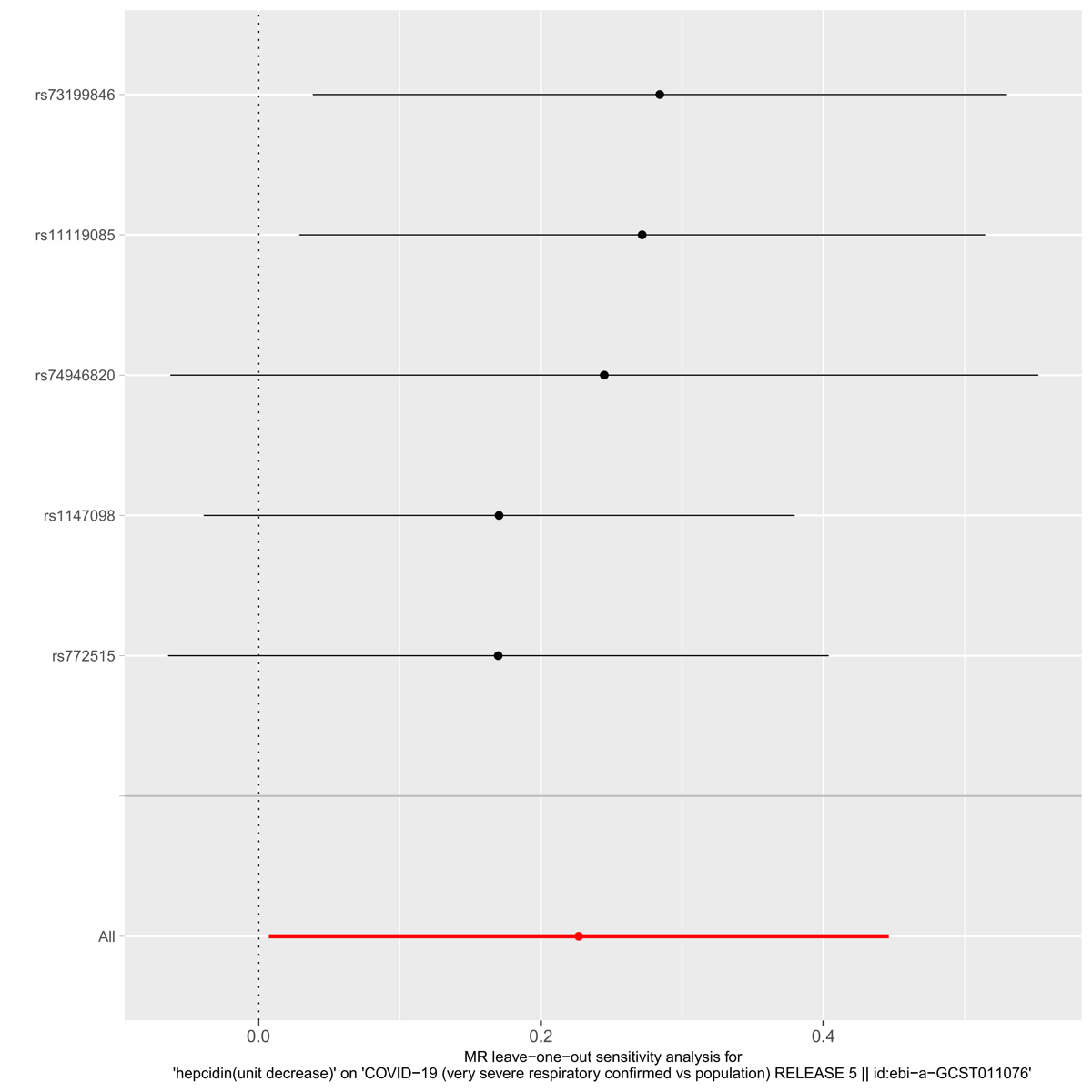


eFig 15. Leave-one-out plots of SNP-specific effects for the associations with decreased hepcidin and COVID-19 (very severe respiratory confirmed vs population) .


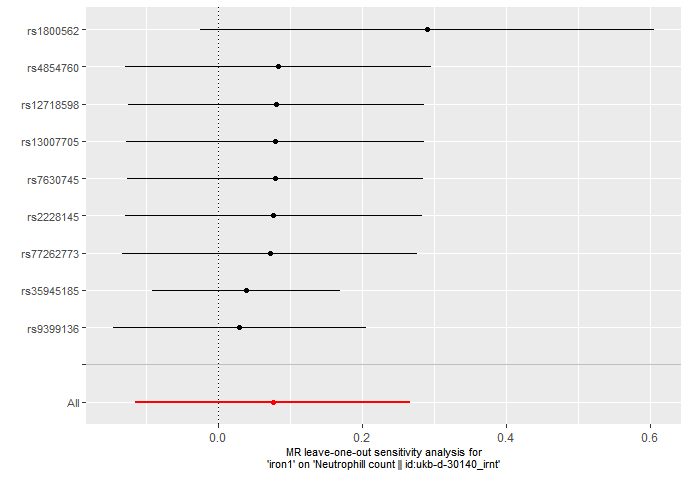


eFig 16. Leave-one-out plots of SNP-specific effects for the associations with increased serum iron and neutrophils.


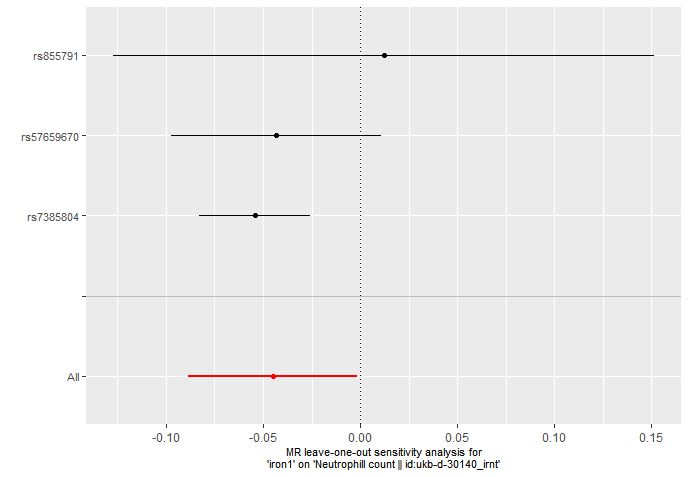


eFig 17. Leave-one-out plots of SNP-specific effects for the associations with decreased serum iron and neutrophils.


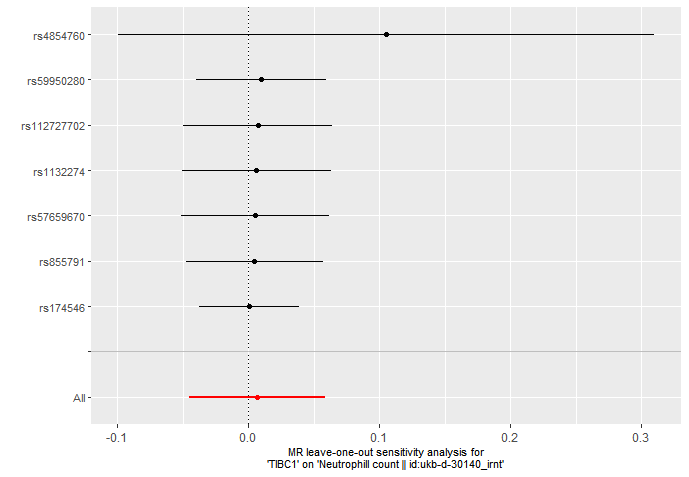


eFig 18. Leave-one-out plots of SNP-specific effects for the associations with increased TIBC and neutrophils.


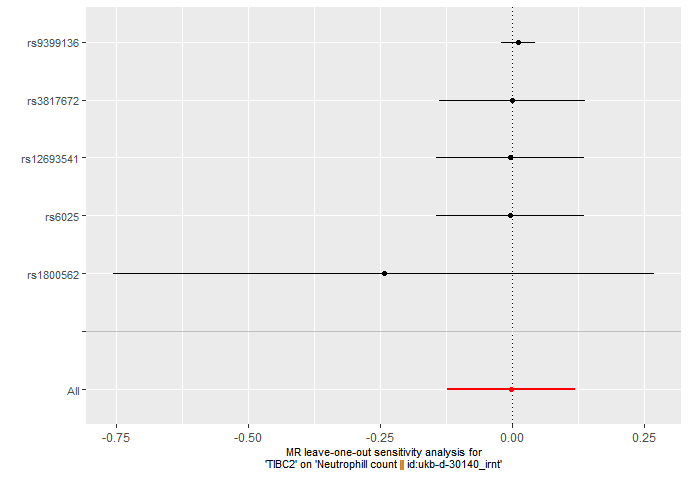


eFig 19. Leave-one-out plots of SNP-specific effects for the associations with decreased TIBC and neutrophils.


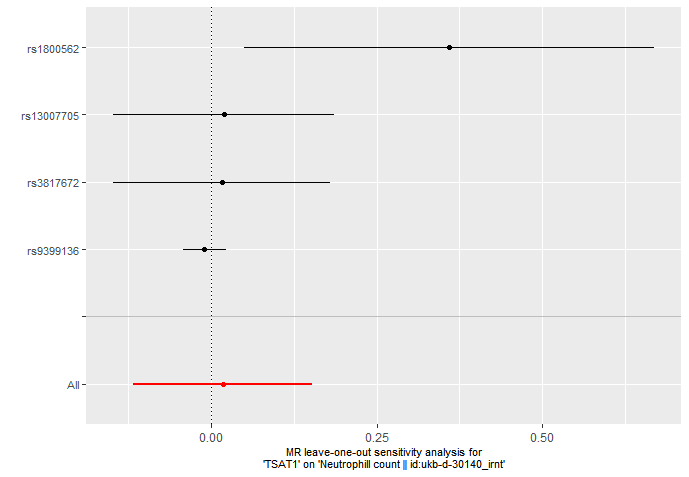


eFig 20. Leave-one-out plots of SNP-specific effects for the associations with increased TSAT and neutrophils.


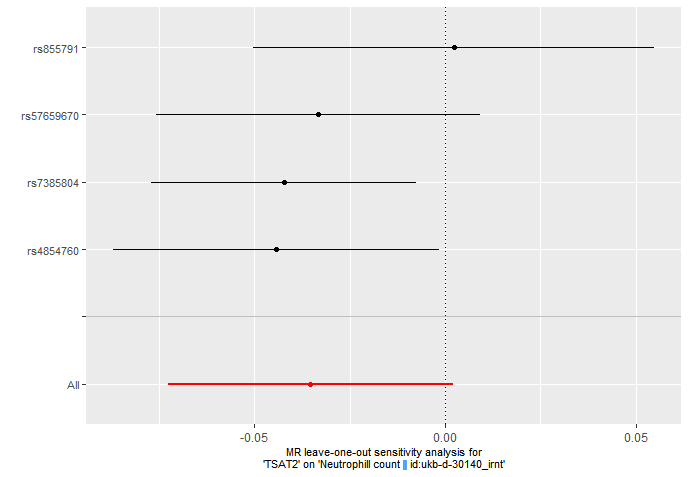


eFig 21. Leave-one-out plots of SNP-specific effects for the associations with decreased TSAT and neutrophils.


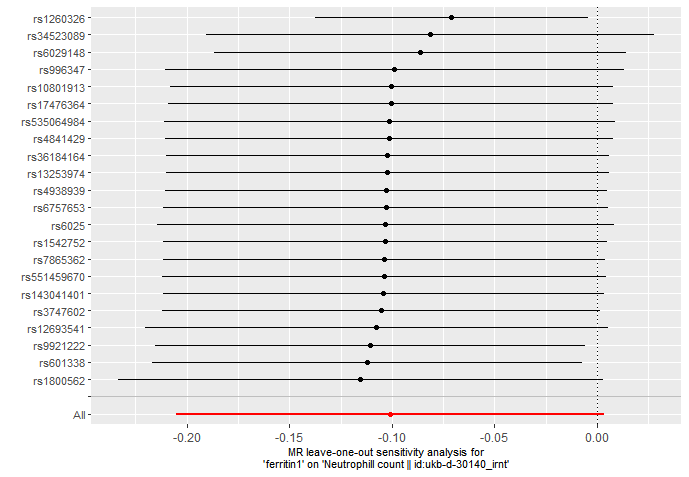


eFig 22. Leave-one-out plots of SNP-specific effects for the associations with increased ferritin and neutrophils.


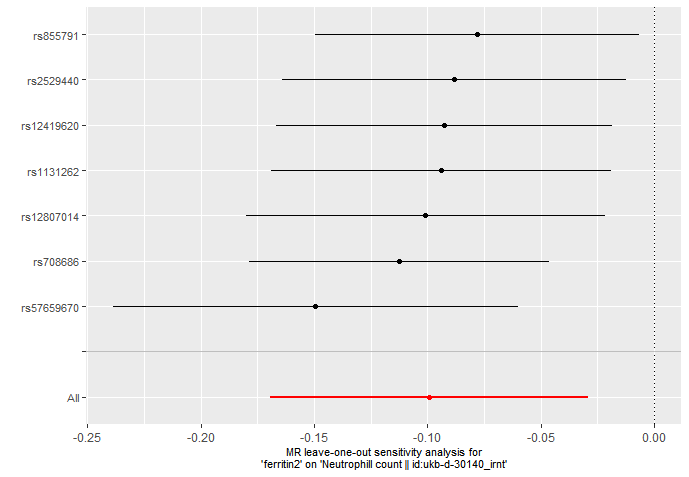


eFig 23. Leave-one-out plots of SNP-specific effects for the associations with decreased ferritin and neutrophils.


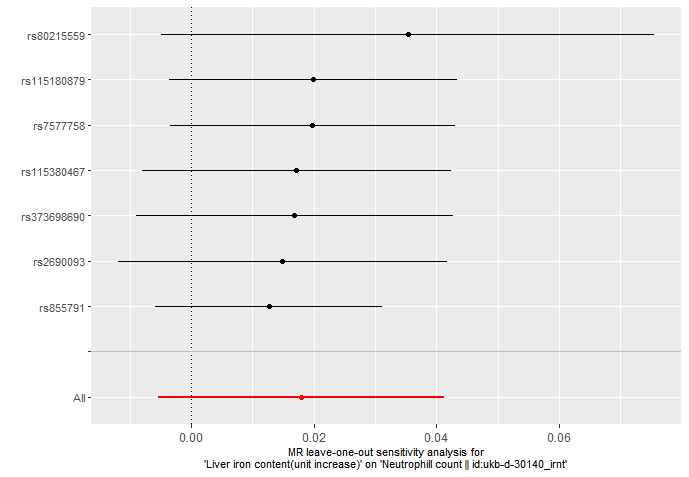


eFig 24. Leave-one-out plots of SNP-specific effects for the associations with increased live iron content and neutrophils.
