# Supplementary material for: Genetic Susceptibility to Causal Relationship Between Iron Metabolism Disorder Involving Immunocytes and Risk of Pneumonia and Sepsis
Source: Food Sci Nutr. 2025 Jun 10;13(6):e70422. doi: 10.1002/fsn3.70422 (PMC12152259; doi:10.1002/fsn3.70422)
Supplement: Supplementary file 3 — Data S3. [file FSN3-13-e70422-s003.zip › fsn370422-sup-0003-DataS3.docx]

**Iron Metabolism Biomarkers in Clinical Practice**

Serum iron reflects bioavailable circulating iron but fluctuates with dietary variation and inflammation. Ferritin, the primary iron storage protein, correlates with body iron stores, but its role as an acute-phase reactant confounded by inflammation or malignancy. Total iron-binding capacity (TIBC) quantifies transferrin’s iron-binding potential, aiding in distinguishing iron deficiency anemia (TIBC↑) from anemia of chronic disease (TIBC↓), yet lacks sensitivity for early iron overload. Transferrin saturation (TSAT, 20–45%), calculated as serum iron divided by TIBC, evaluates functional iron supply, with thresholds <20% indicating deficiency and >45% suggesting overload, though hemolysis or acute infections may confound results. Lactotransferrin, an iron-binding antimicrobial protein, modulates nutritional immunity but has limited routine clinical utility due to non-standardized assays. Liver iron content, measured via MRI or biopsy, is the gold standard for assessing iron overload but is invasive and costly. Collectively, these biomarkers offer complementary insights: ferritin and TSAT are effective for screening, while liver iron quantification confirms diagnoses. Key limitations include inflammation-driven interference (ferritin, TSAT) and insufficient dynamic range for early-stage iron disorders (TIBC). Integrating multiple markers mitigates individual weaknesses, enhancing diagnostic accuracy for conditions like hereditary hemochromatosis or anemia of chronic disease. The clinical reference ranges and change trends of iron metabolism biomarkers in anemias see the following List 1 and List 2

List 1. Clinical Reference Ranges of Iron Metabolism Biomarkers.

| Biomarker | Reference Range |
| --- | --- |
| Serum Iron | Men 60–170 μg/dL; Women 50–150 μg/dL |
| Ferritin | Men 30–300 μg/L; Women 15–150 μg/L |
| TIBC | 240–450 μg/dL |
| TSAT | 20–45% |
| Lactotransferrin | Serum 0.5–2.0 mg/L |
| Liver iron content | MRI <1.8 mg/g; Biopsy <7 mg/g |

List 2. Change Trends of Iron Metabolism Biomarkers in Anemias

| Biomarker | IDA | ACD | AI | IO |
| --- | --- | --- | --- | --- |
| Serum Iron | ↓↓  (<30 μg/dL) | ↓ or NM | ↓  (hypoferremia) | ↑↑ (>200 μg/dL) or ↓ (transfer into cells) |
| Ferritin | ↓↓  (<30 μg/L) | ↑  (100–300 μg/L) | ↑  (acute-phase response) | ↑↑↑  (>500 μg/L) |
| TIBC | ↑↑  (>400 μg/dL) | ↓↓  (<250 μg/dL) | ↓ | ↓ or NM |
| TSAT | ↓↓  (<10%) | NM or ↓ (15–20%) | ↓  (transient) | ↑↑ (>55%) |
| Lactotransferrin | NM or ↑ | ↓  (chronic inflammation) | ↑↑  (antimicrobial response) | ↓  (iron redistribution) |
| Liver iron content | NM | NM or ↑ | NM or ↑ | ↑↑↑  (>7 mg/g) |

Note: IDA, Iron Deficiency Anemia; ACD, Anemia of Chronic Disease; AI, Anemia of infection caused by Acute Infection (Nutritional Immunity); IO, Iron Overload; NM, normal.
